# A symbiotic bacterium of Antarctic fish reveals environmental adaptability mechanisms and biosynthetic potential towards antibacterial and cytotoxic activities

**DOI:** 10.3389/fmicb.2022.1085063

**Published:** 2023-01-13

**Authors:** Yu Xiao, Fangfang Yan, Yukun Cui, Jiangtao Du, Guangzhao Hu, Wanying Zhai, Rulong Liu, Zhizhen Zhang, Jiasong Fang, Liangbiao Chen, Xi Yu

**Affiliations:** ^1^Shanghai Engineering Research Center of Hadal Science and Technology, College of Marine Sciences, Shanghai Ocean University, Shanghai, China; ^2^International Research Center for Marine Biosciences, Ministry of Science and Technology, Shanghai Ocean University, Shanghai, China; ^3^Ocean College, Zhoushan Campus, Zhejiang University, Zhoushan, China

**Keywords:** Antarctic fish, symbiotic bacteria, *Serratia*, genome, bioactive metabolites

## Abstract

Antarctic microbes are important agents for evolutionary adaptation and natural resource of bioactive compounds, harboring the particular metabolic pathways to biosynthesize natural products. However, not much is known on symbiotic microbiomes of fish in the Antarctic zone. In the present study, the culture method and whole-genome sequencing were performed. Natural product analyses were carried out to determine the biosynthetic potential. We report the isolation and identification of a symbiotic bacterium *Serratia myotis* L7-1, that is highly adaptive and resides within Antarctic fish, *Trematomus bernacchii*. As revealed by genomic analyses, Antarctic strain *S. myotis* L7-1 possesses carbohydrate-active enzymes (CAZymes), biosynthetic gene clusters (BGCs), stress response genes, antibiotic resistant genes (ARGs), and a complete type IV secretion system which could facilitate competition and colonization in the extreme Antarctic environment. The identification of microbiome gene clusters indicates the biosynthetic potential of bioactive compounds. Based on bioactivity-guided fractionation, serranticin was purified and identified as the bioactive compound, showing significant antibacterial and antitumor activity. The serranticin gene cluster was identified and located on the chrome. Furthermore, the multidrug resistance and strong bacterial antagonism contribute competitive advantages in ecological niches. Our results highlight the existence of a symbiotic bacterium in Antarctic fish largely represented by bioactive natural products and the adaptability to survive in the fish living in Antarctic oceans.

## Introduction

The unique thermal and geographic isolation of the Antarctica continent creates some of the coldest and yet stable environmental conditions in the world’s oceans (Tercero and Place, 2020; Giuliani et al., 2021). The high latitude Antarctic fishes, inhabiting the world’s coldest environment, are ideal model organisms for decoding novel traits that may arise during the evolution in millions of years (Obrien, 2016). At present, the dominant species in Antarctic Ocean are the suborder Notothenioidei belonging to the Perciformes, including the *Trematomus bernacchii*, *Cottoperca gobio*, *Gymnodraco acuticeps*, etc. (Eastman, 2004). Given the ecological, morphological, and physiological diversification of the Antarctic fishes, one may anticipate that their symbiotic microbial communities have also evolved to accommodate the various trophic lifestyles of the hosts (Ward et al., 2009). Symbiotic bacteria have many roles in the host, most of which are correlated with host nutrition, immunity, development, and resistance against the cold environment (Sedláček et al., 2016; Song et al., 2016). The symbiotic microbiota contributes to the protective effects both through the direct influence *via* microbial metabolites and indirect effects of microbiota on the host immune system (Li et al., 2016). On the other hand, the symbionts develop extended survival capability to compete with other microbes and the external environment (Dixon, 2001).

An increasing number of studies have focused on the microbial potential to keep the functional genes and biosynthesize secondary metabolites as a reservoir (Xuan et al., 2015). Secondary metabolites are a major source of natural products, which act as an arsenal of antibiotics. These structurally unique natural products display potent biological activity, including antitumor, antimicrobial, antiviral, and antiparasitic activities, mediated by versatile biosynthetic gene clusters (Wang et al., 2020). Microbial natural products represent a huge and largely untapped resource of unique chemical structures that have been optimized by evolution and are produced for communication and in response to changes in microbial habitats, including environmental stresses (Gutierrez and Gonzalez, 2012). The close association between the symbiotic microbiota with these compounds could be important in bacterial antagonism under the high competition pressure. The attributes that characterize the fish symbiotic bacteria is still not well understood. Knowledge of bacterial species, associated with the potential production of bioactive compounds, is important to clarify the adaptation strategies of symbiotic microbiota in fish and benefit for extensive use in the pharmaceutical industry.

To understand microbial diversity and gene functions, multi-omics combined with taxonomic profiling and functional analyses has become one of the most effective approaches. A series of microbial metagenomic analysis from Antarctic zone were conducted for their ecological role and capabilities to influence the host. In the Antarctic fish gut microbiome, Proteobacteria, Actinobacteria and Firmicutes were the dominant phyla, and *Vibrio*, *Serratia*, *Aeromonas* were the dominant genera (Cormack and Fraile, 1990; Maccormack and Fraile, 1991; Ward et al., 2009; Sedláček et al., 2016; Song et al., 2016). However, only culture methods and 16S rRNA sequencing have been used in past studies to identify the composition of Antarctic fish symbiotic microbes. The lack of functional studies of symbiotic microbes hinders the understanding of microbial adaptation in the fish. In particular, natural product analyses of specific groups of animal symbiotic microbiota within such extreme environments is rarely performed.

Here we report the isolation and identification of *Serratia myotis* L7-1, a symbiotic bacterium of Antarctic fish, which showed high adaptation to the living environment. The detailed taxonomic analysis and genomic annotation of the sequenced symbiotic bacteria of a high-latitude benthic fish- *Trematomus bernacchii*, were provided. The functional analysis of the genome indicated the important roles of *S. myotis* L7-1 in microbial metabolism, antibiotic resistance, energy uptake, and health of the fish. The analyses of biosynthetic gene clusters and the bioactivity assay displayed that *S. myotis* has the capability of antibiotic production. Importantly, *S. myotis* L7-1 produced bioactive secondary metabolites to recruit weaponry during microbe-microbe communications, such as serranticin that effectively inhibits the growth of aquatic pathogens *Aeromonas hydrophila* and *Edwardsiella piscicida*, which may improve the competitiveness of *S. myotis* L7-1.

## Materials and methods

### Sample collection and preparation

Fish *T. bernacchii* (66.8 g) was collected by ice fishing from the Antarctic China Zhongshan Station (69°22′27”S, 76°22′20″E) in February 2020. After the morphological identification, the fish was characterized using DNA barcoding of cytochrome oxidase subunit I (COI). The fresh fish was sealed and stored in sterile plastic bags immediately at 4°C for subsequent experiments. The intact fish abdominal contents were aseptically dissected from the fish, and 2 ml were taken into a centrifuge tube to investigate the composition and function of symbiotic bacteria.

### Symbiotic bacteria isolation

The fish abdominal contents were diluted with 3.4% NaCl to make dilutions. Each dilution (200 μl) was plated on the surface of the isolation medium. The media used for isolation were luria-bertani agar (LB), nutrient agar (NA), tryptose soya agar (TSA), reinforced clostridium medium (RCM), modified artificial seawater medium (MASM), potato dextrose agar (PDA), yeast malt glucose (YMG), minimal medium (MM; Supplementary Table S1), brain heart infusion agar (BHIA, Hopebio, Qingdao, China), 2216E agar (Acmec, Shanghai, China). The plates were incubated at 20°C for 3 days and bacterial growth were detected. The distinct colonies were picked up and purified *via* repeated streaking (at least three times) on the isolation medium, which then were maintained in 50% glycerol and preserved at −80°C for further study.

### 16S rDNA cloning and sequencing for bacterial identification

Genomic DNA was extracted from bacteria cells using the Rapid Bacterial Genomic DNA Isolation Kit (Sangon, Shanghai, China) according to the manufacturer’s instructions. The 16S rDNA genes were amplified by PCR using 27F (5’-AGAGTTTGATCCTGGCTCAG-3′) and 1492R (5’-GGTTACCTTGTTACGACTT-3′) primers. The amplified DNA sequences were analyzed by Azenta Life Science Co., Ltd. for 16S rDNA sequencing, and the 16S sequences were aligned and identified against existing sequences in the NCBI database using the BLAST program. Further, the nucleotide sequences of the isolates were aligned with closely related sequence using Mega software (Kumar et al., 2008) and a phylogenetic tree was constructed to show the relationship between the isolates and the reference strains.

### DNA extraction and 16S rRNA deep sequencing

Genomic DNA was extracted using the E.Z.N.A.^®^ soil DNA Kit (Omega Bio-tek, Norcross, GA, United States) based on the manufacturer’s protocol. The hyper-variable regions V3-V4 of the 16S rRNA gene were amplified using a universal primer set 338F (5’-ACTCCTACGGGAGGCAGCAG-3′) and 806R (5’-GGACTACHVGGGTWTCTAAT-3′). Sequencing of the 16S amplification was performed by Majorbio Bio-Pharm Technology Co. Ltd. (Shanghai, China) on an Illumina MiSeq PE300 platform (Illumina, San Diego, United States) with two paired-end read cycles of 300 bases each. The raw 16S rRNA gene sequencing reads were demultiplexed, quality-filtered by fastp version 0.20.0 (Chen et al., 2018) and merged by FLASH version 1.2.7 (Magoč and Salzberg, 2011). Operational taxonomic units (OTUs) with 97% similarity cutoff (Stackebrandt and Goebel, 1994; Edgar, 2013) were clustered using UPARSE version 7.1 (Edgar, 2013), and chimeric sequences were identified and removed. The taxonomy of each OTU representative sequence was analyzed by RDP Classifier version 2.2 (Wang et al., 2007) against the 16S rRNA database using confidence threshold of 0.7.

### Genomic DNA preparation and whole-genome sequencing

The Wizard® Genomic DNA Purification Kit (Promega) was used for genomic DNA extraction according to the manufacturer’s protocol. We utilized the Nanopore (Oxford Nanopore) and Illumina HiSeq X Ten sequencing platforms to perform whole-genome shotgun sequencing. The sequencing service was provided by Majorbio Bio-Pharm Technology Co. Ltd. (Shanghai, China).

### *De novo* genome assembly and genome annotation

The original image data was transferred into sequence data *via* base calling, which was defined as raw data or raw reads and saved as FASTQ file. A statistic of quality information was applied for quality trimming, by which the lower quality data was removed to form clean data. The reads were assembled into a contig using hierarchical genome assembly process (HGAP) and canu. The last circular step was checked and finished manually, generating a complete genome with seamless chromosomes and plasmids. Finally, error correction of the assembly results was performed using the Illumina reads. Glimmer (Delcher et al., 2007) was used for CDS prediction, tRNA-scan-SE (Chan and Lowe, 2019) was used for tRNA prediction and Barrnap was used for rRNA prediction. The predicted CDSs were annotated from NR, Swiss-Prot (Bairoch and Apweiler, 2000), Pfam (Finn et al., 2013), GO (Consortium G O, 2004), COG (Lars Juhl et al., 2008) and KEGG (Ogata et al., 1999) database using sequence alignment tools such as BLAST (Ye et al., 2006), Diamond (Neshich et al., 2005) and HMMER (Potter et al., 2018). Briefly, each set of query proteins were aligned with the databases, and annotations of best-matched subjects (e-value <10^−5^) were obtained for gene annotation.

### Mass culture of Strain *Serratia myotis* L7-1

Colonies of L7-1 were inoculated into 250 ml of sterile LB medium and then incubated at 20°C for 24 h on a rotary shaker (180 rpm) to produce seed broth. The seed broth (12 ml) was transferred into a 500 ml Erlenmeyer flask, containing fresh rice solid medium (rice 40 g, sterile seawater 60 ml). The flasks were in a state for cultivation at 20°C for 30 days. A total of 50 flasks were prepared for this study. The rice solid culture of L7-1 was extracted with ethyl acetate three times and evaporated dry by rotary evaporator to give a crude extract for subsequent analysis.

### Extraction and isolation of compounds

The crude extract (16.7 g) was fractionated by a silica gel column eluting with mixed solvents of PET/EtOAc (2/1, 1/1, 1/2, 1/5, 1/10, v/v), and EtOAc/MeOH (5/1, 1/1, 1/5, v/v) to furnish eight fractions (Fr-1-Fr-8) based on the results of TLC analysis. Fr. 4 (3.4 g), was further purified by Octadecyl-functionalized silica gel column (ODS, Cosmosil 75C_18_-Prep) with MeOH/H_2_O (3,7, 1,1, 7:3, 1:0, v/v) to afford four subfractions (Fr. 4.1–4.4). Compound 1 (61 mg, t_R_ 20 min, MeOH/H_2_O, 30/70) was purified from Fr. 4.3 by Agilent 1,260 HPLC using a column (Agilent Zorbax SB-C_18_, 250 × 9.4 mm, 5 μm; flow rate: 1 ml/min; UV detection: 210 nm). HPLC and analytic grace solvents used for this study were purchased from Sinopharm Chemical Reagent Co. Ltd. (Shanghai, China).

### Identification of compounds structures

Optical rotation was recorded on a RUDOLPH Autopol I Automatic polarimeter (Rudolph Research Analytical). NMR spectra were acquired on a Bruker 500 spectrometer or a JEOL 600 spectrometer using the standard programs and acquisition parameters and the chemical shifts were expressed in δ (ppm) relative to DMSO-d6 (δC 39.5 and δH 2.50).

### Antibacterial assay of crude extracts

The indicator bacteria used in the bacteriostatic circle experiment were provided by Shanghai Rainbowfish Company, including *Chromobacterium violaceum* ATCC 12472, *Salmonella choleraesuis*, *Mycobacterium smegmatis* MC2155, *Escherichia coli* MG1655, *Enterococcus faecalis* FA2-2, *Staphylococcus aureus* ATCC25923. Cultures of six indicator bacteria (with an OD of approximately 0.5) were inoculated in LB medium in an amount of 100 μl. The sterilized circular filter paper sheets were attached to the LB. Dissolve 10 mg of the crude extract in 1 ml of methanol. Then 6 μl of the crude extracted metabolites and negative control (methanol) were dropped onto the circular filter paper sheet, after incubated at 37°C for 12 h. Antibacterial bioactivity was evaluated by measuring the area of the zones inhibition and calculating the inhibition rate, which was measured with Image J.

### Antibacterial assay of serranticin

The antibacterial activities of serranticin inhibiting the growth of *C. violaceum*, *S. choleraesuis*, *M. smegmatis*, *E*. *coli*, *E. faecalis*, *S.aureus E. tarda*, and *A. hydrophila* were determined by the micro-broth dilution method (Xin et al., 2012). Gentamicin was used as a positive control and DMSO was used as a negative control.

### Cell lines and culture

Human lung cancer NCI-H460, human breast carcinoma MCF-7 and MDA-MB-231 cell lines were obtained from the National Collection of Authenticated Cell Cultures (Shanghai, China). All cells were incubated at 37°C in a humidified incubator with 5% CO_2_ incubator. Cells after the third generation were used for experiment.

### Assay for anti-proliferation

The assay using 3-(4,5-dimethylthiazole-2-yl)-2,5-dip-. henyltetrazolium bromide (MTT) against the MDA-MB-231, NCI-H460, and MCF7 cell lines (Huang et al., 2008). Cells (3 × 10^3^ per well for cancer cell lines) were seeded with 200 μl of medium in 96-well plates. we dissolved the test sample in dimethyl sulfoxide (DMSO) to final concentrations of 0.78, 1.56, 3.13, 6.25, 12.5, 25, 50, and 100 μM in each well in assays against the MDA-MB-231, NCI-H460 and MCF7 cell lines. The blank control treatment used an equivalent volume of DMSO.

### Identification of the serranticin biosynthetic cluster

To find the gene cluster responsible for serranticin biosynthetic, we first download the whole genome sequence of *Serratia plymuthica* V4 (Accession number NZ_CP007439.1) and searched for the genome sequence of synthetic compound serratiochelin.

### K-B agar diffusion assay for drug sensitivity

Drug sensitivity tests were performed on seven common clinical antibiotics *via* the K-B agar diffusion method. Cultures of *S. myotis* L7-1 (with an OD of approximately 0.5) were inoculated in LB medium in an amount of 100 μl. The sterilized circular filter paper sheets were attached to the LB. Then 6 μl of the antibiotics (1 mg/ml) and negative control (LB medium) were dropped onto the circular filter paper sheet, after incubated at 37°C for 18–24 h. Antibacterial assay was evaluated by measuring the diameter of the zone inhibition.

## Results

### *Serratia* bacteria are the core isolated symbionts of *Trematomus bernacchii*

**Figure 1:**
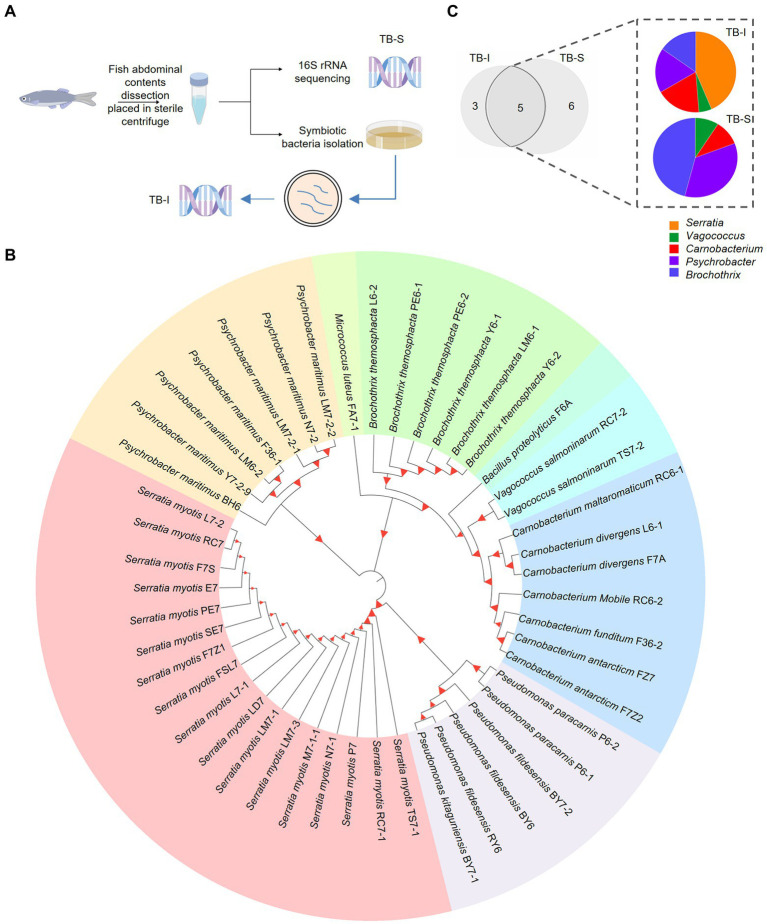
Isolation of the symbiotic *Serratia* bacteria of fish. **(A)** Workflow of the isolation and identification of symbiotic bacteria in *T. bernacchii* using *16S* rRNA sequencing and culture method (TB-I and TB-S). www.figdraw.com. **(B)** Phylogenetic tree showed the kinship and composition of 47 distinct bacterial strains isolated from *T. bernacchii*. Itol.embl.de. **(C)** Composition and relative abundance of the genera shared between the two methods. Origin 2022.

The symbiotic microbiota is typically beneficial or neutral during the multi-species interactions (Moreau, 2020). To explore the role of symbionts, we designed and performed two assays to identify the composition of the fish symbiotic microbiota (Figure 1A). The bacteria enrichment samples of Antarctic fish *Trematomus bernacchii* were harvested. Symbiotic bacteria of *T. bernacchii* were isolated using the culture method (TB-I). A total of 47 distinct bacterial isolates were selected for 16S rDNA sequence identification (Figure 1B). Phylogenetic analysis revealed that a few taxa were predominated among these isolated strains, and the dominant bacterial species of *T. bernacchii* sample belonged to *Serratia*. Whether it was a nutrient-rich medium or a low-nutrient medium, *Serratia* was the abundant species (36.2%) and maintained growth under different nutrient conditions (Supplementary Figure S1A; Supplementary Tables S1, S2).

We also extracted DNA from *T. bernacchii* abdominal contents and deep sequencing of 16S rRNA (TB-S) revealed a total of 11 bacterial species (Supplementary Figure S1B). There were large differences in the bacterial compositions displayed by the two methods. Despite the differences in bacteria composition, the two methods shared five bacterial genera, *Serratia*, *Vagococcus*, *Carnobacterium*, *Psychrobacter*, and *Brochothrix* (Figure 1C). These 3 unique bacteria genera in TB-I included *Pseudomonas*, *Micrococcus*, and *Bacillus*. These 6 unique bacteria genera in TB-S included *norank-f-Bacillaceae*, *Photobacterium*, *Corynebacterium*, *Jeotgalicoccus*, *Aerococcus*, and *Paracoccus* (Figure 1C). Notably, the relative abundances of genus *Serratia* varied considerably among the shared bacterial symbionts in the two compositions. *Serratia* (0.02%) was the least abundant bacteria in the 16S rRNA sequencing (TB-S). Whilst *Serratia* (43.6%) was the most abundant bacteria in the culture method (Figure 1C). Moreover, results of the different culture plates showed that *Serratia* species had the highest proportion among all the isolated strains from the various media. The growth assay determined that *Serratia* kept the highest growth viability in minimal nutrient media (MN; Supplementary Figure S2). The obtained results led us to assume that *Serratia* sp. was highly adaptive, and able to tolerate a range of nutrient conditions.

### *Serratia myotis* L7-1 has the potential to biosynthesize bioactive compounds

**Figure 2:**
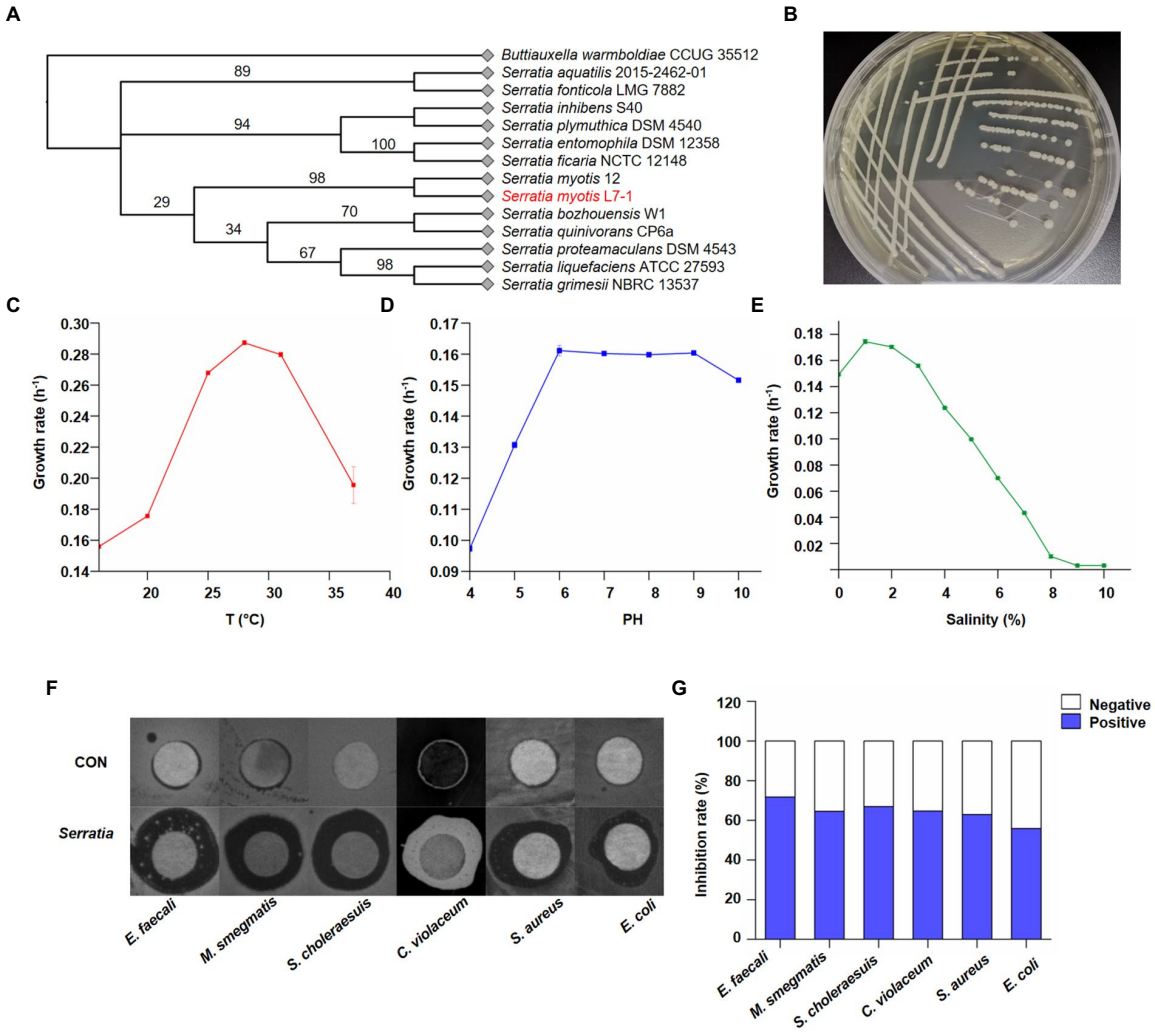
Identification of *Serratia myoti*s as the core symbiotic bacteria. **(A)** Neighbor-joining tree based on nearly complete *16S* rDNA gene sequences of *Serratia myotis* L7-1 and close taxa of the genus *Serratia*. *Buttiauxella warmboldiae* CCUG 35512 was used as an outgroup. Itol.embl.de. **(B)** Macroscopic images showed the morphology of *S. myotis* L7-1 colony. **(C)** Growth rate of *S. myotis* L7-1 in different temperature (*n* = 3). **(D)** Growth rate of *S. myotis* L7-1 in different PH values (*n* = 3). **(E)** Growth rate of *S. myotis* L7-1 in different salinity (*n* = 3). Origin 2022. **(F)** Zones of inhibition of the metabolites produced by *Serratia*. **(G)** *In-vitro* antimicrobial activity of the metabolites produced of the indicated *Serratia* strain. Origin 2022.

A total of 17 strains of symbiotic bacteria were identified as *Serratia myotis* based on 16S rDNA sequence (Supplementary Table S2), with similarity between 99.50 to 99.86%. To determine the relationship between the 17 strains of *S. myotis*, we identified the homologous sequence between them using DNA star, with interspecific similarity between 99.40 to 100% (Supplementary Table S2). Based on the high similarity of sequences, *S. myotis* L7-1 was selected as a representative randomly for further studies (Figure 2A). The following description of *S. myotis* L7-1 was based on data reported previously. *S. myotis* is gram-stain-negative, facultative anaerobic, non-spore-forming, rod-shaped cells, 1–1.3 mm in length and 0.5–0.6 mm in diameter, and motile by subpolar flagella (García-Fraile et al., 2015). In our cultivation, *S. myotis* L7-1 colony morphology on LB agar medium was smooth, circular, convex and transparent, with a diameter of 1.5 to 2.0 mm after 48 h of growth at 20°C (Figure 2B). Optimal growth was observed at 25–31°C (Figure 2C; Supplementary Figure S3A), pH 6–9 (Figure 2D; Supplementary Figure S3B) and with 0–3% NaCl (Figure 2E; Supplementary Figure S3C). Type strain is 12 T (=CECT 8594^T^ = DSM 28726^T^). 16S rDNA sequence accession number (GenBank) is KJ739884 (García-Fraile et al., 2015).

Chemical agents in the living environment of symbiotic microbiota exhibit a broad range of biological activities (Lu et al., 2016). Our next question was whether the natural products of *Serratia* sp. were of biological meaning as the antibiotics. To confirm their bioactivities, crude extracts from the isolates were tested for antimicrobial activity toward six bacterial species with the disk diffusion method. We found that the crude extract of *S. myotis* could consistently inhibit the growth of pathogenic bacteria (Figure 2F). Whereas, these of other isolated bacterial species from the same sample showed no significant antibacterial activity (Supplementary Figure S4). Next, we further investigated the effectiveness of *S. myotis* L7-1. The crude extract of *S. myotis* exhibited antibacterial activity against *Enterococcus faecalis*, *Mycobacterium smegmatis*, *Salmonella choleraesuis*, *Chromobacterium violaceum*, *Staphylococcus aureus* and *Escherichia coli* at 10 mg/ml, with inhibition rate of 71.8, 64.6, 66.9, 64.7, 63.0, 55.9% (Figure 2G), respectively. It seems that, *S. myotis* L7-1 could be a natural reservoir of bioactive secondary metabolites.

### *Serratia myotis* L7-1 genome organization and genomic traits related to SMs production and adaptation

**Figure 3:**
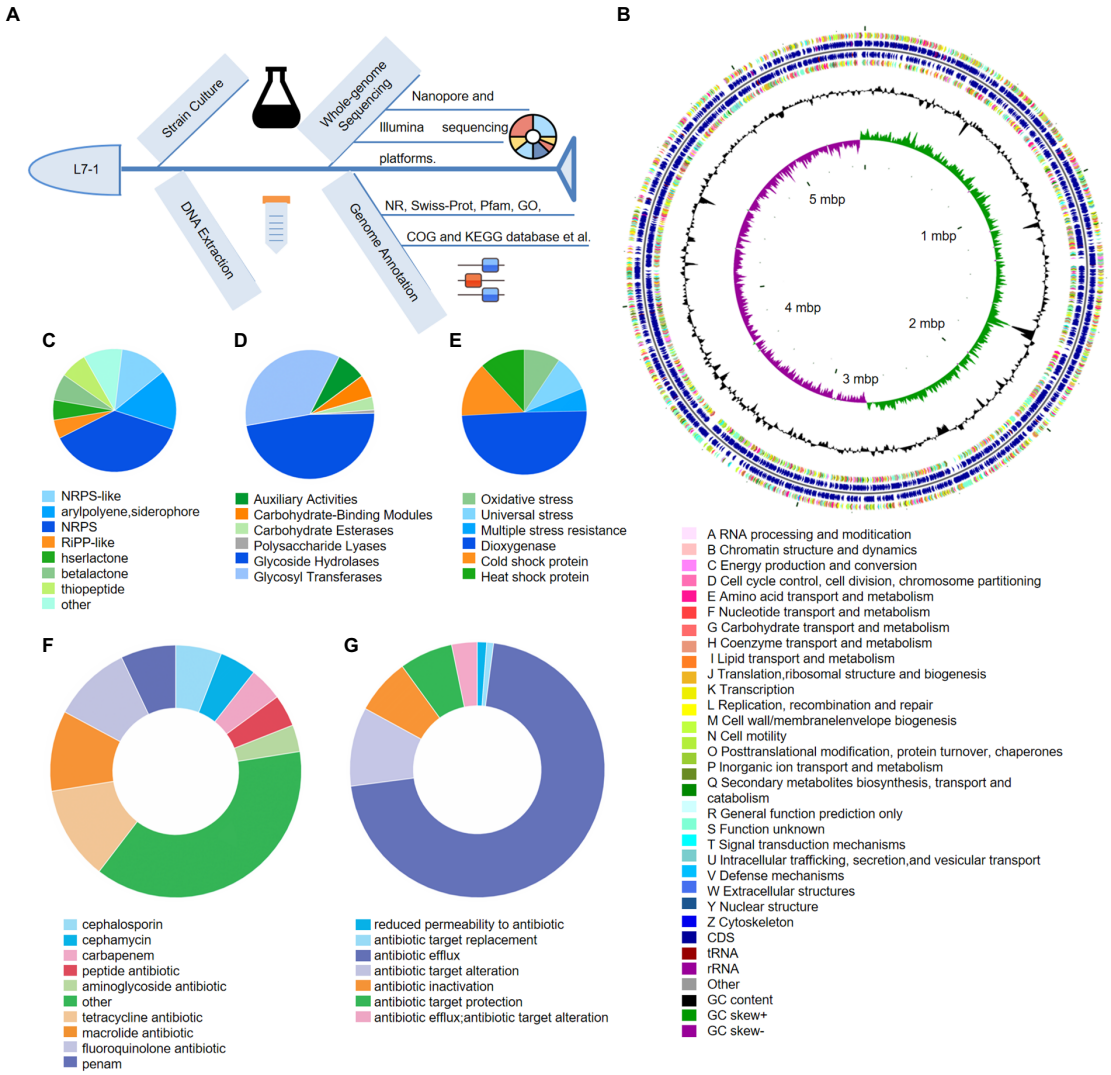
Genomic annotation and traits of *S. myotis* L7-1. **(A)** Workflow of the whole-genome sequencing of *S. myotis* L7-1 strain using Nanopore sequencing. **(B)** Circular map of chromosome of *S. myotis* L7-1. From outside to inside ring 1 and 4 depicts CDS in the positive strand and negative strand, different colors indicate different COG functional classifications. Ring 2 and 3 depicts CDS, tRNA and rRNA in the positive strand and negative strand. Ring 5 and 6 represents GC content and GC skew, respectively. cloud.majorbio.com. **(C)** Composition and relative abundance of secondary metabolic synthesis gene cluster genes of *S. myotis* L7-1. **(D)** Composition and relative abundance of CAZyme genes of *S. myotis* L7-1. **(E)** Composition and relative abundance of environmental adaptation genes of *S. myotis* L7-1. **(F)** Composition and relative abundance of antibiotic resistance genes of *S. myotis* L7-1. **(G)** Composition and relative abundance of antibiotic resistance mechanism of *S. myotis* L7-1. Origin 2022.

The whole genomic annotation was performed to decipher the functional genes of *S. myotis* in the Antarctic fish (Figure 3A). The sequences obtained from *S. myotis* L7-1 were assembled into one chromosome and four plasmids of size 5,503,952 bp (Chr 1; Figure 3B), 110,419 bp (pA; Supplementary Figure S5A), 49,145 bp (pB; Supplementary Figure S5B), 38,106 bp (pC; Supplementary Figure S5C), 3,223 bp (pD; Supplementary Figure S5D), with a GC content of 55.06%, and 5,272 genes with coding sequences. A total of 85 tRNA, 22 rRNA and 120 sRNA were detected. A total of 5,357 genes were predicted to display COG functional annotation, including 2,096 transport and metabolism genes, 2,550 cellular process and signal transduction genes, 526 information storage and processing genes, and 185 functionally unknown genes.

Functional classification of the microbial genes revealed the prevalence of genes for the metabolic system, including the putative biosynthetic gene clusters (BGCs) and carbohydrate-active enzymes (CAZymes). We identified a total of 285 BGCs associated genes and assigned secondary metabolic pathways using antiSMASH (Kai et al., 2017; Figure 3C). The results revealed the presence of 10 gene clusters encoding biosynthesis of secondary metabolites (SMs), including three non-ribosomal peptide-synthetase (NRPS; 37.5%), a hserlactone (5.3%), a NRPS-like (12.3%), a betalactone (7%), an arylpolyene, siderophore (15.8%), a thiopeptide (7%), a Ribosomally synthesized and post-translationally modified peptides-like (RiPP-like; 4.9%), and other (10.2%) gene clusters (Supplementary Figure S6). The percentage values mean the proportion of the related genes in the total BGCs genes. The abundant biosynthetic genes and related clusters indicated the important roles of versatile SMs production in *S. myotis* L7-1, as an untapped resource of undiscovered molecules. CAZymes analysis (Cantarel et al., 2009) identified 122 proteins belonging to the families of structurally-related catalytic and carbohydrate-binding modules (or functional domains) of enzymes that degrade, modify or create glycosidic bonds. Glycoside hydrolase (GH) family (47.5%) and glycosyl transferases (GTs; 35.2%) were the most abundant proteins in the CAZymes (Figure 3D).

Next, we showed *S. myotis* L7-1 harbored 85 genes associated with the “stress response” and linked to adaptation to extreme environments. Among these genes, universal stress genes (9.4%), multiple stress resistance genes (5.9%), dioxygenase genes (49.4%) and genes related to “oxidative stress” (9.4%), might assist the strain to cope with oxidative stress in seawater caused by the Antarctic environment. Cold shock protein genes (14.1%) and heat shock protein genes (11.8%) would facilitate the strain to survive in Antarctic temperatures (Figure 3E). Another interesting finding related with environmental adaptation was that several antibiotic-resistance genes (ARGs) and multiple antibiotic resistance mechanisms were predicted in the genome of *S. myotis* L7-1, by exploring the pan-genome analysis from the CARD database (Jia et al., 2016). The majority of ARGs contribute to encode the cephalosporin (5.9%), cephamycin (4.7%), carbapenem (4.3%), peptide antibiotic (4.1%), aminoglycoside antibiotic (3.4%), tetracycline antibiotic (12%), macrolide antibiotic (10.3%), fluoroquinolone antibiotic (10.1%), penam (7.1%) and others (38%; Figure 3F; details in Supplementary Table S3). Furthermore, antibiotic resistance mechanism included the reduced permeability to antibiotic (1.2%), antibiotic target replacement (0.9%), antibiotic efflux (70.9%), antibiotic target alteration (10%), antibiotic inactivation (7.1%), antibiotic target protection (6.8%), and antibiotic efflux, antibiotic target alteration (3.2%; Figure 3G). These results suggest that *S. myotis* had the ability to resist several antibiotics, and the ARGs possibly facilitated the competitiveness and colonization of bacteria in the extreme Antarctic environments.

### *Serratia myotis* L7-1 exhibits the putative adaptability mechanisms *via* the comparative genomics

**Figure 4:**
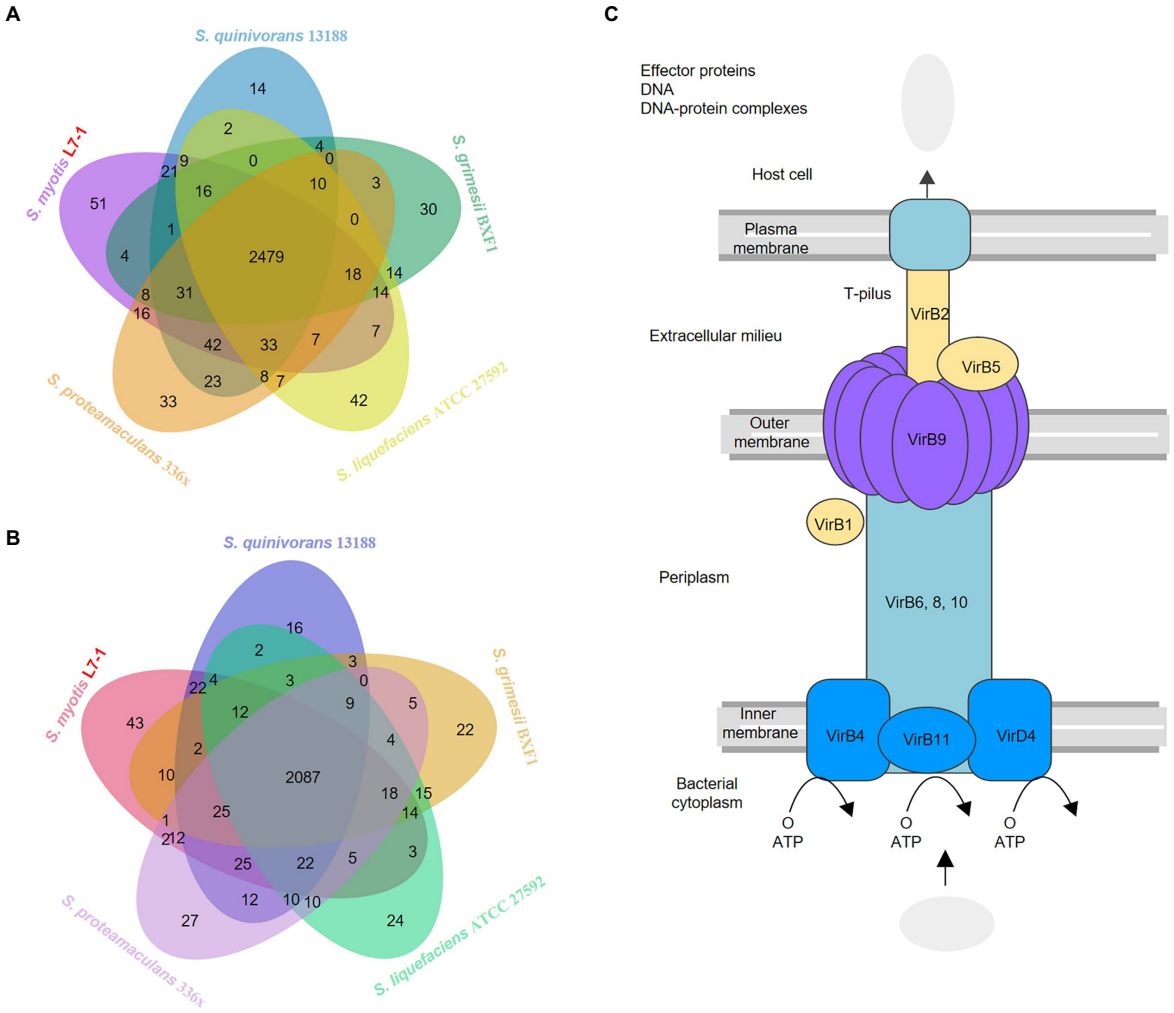
Comparative genomics indicates the specific adaptability mechanisms **(A)** Venn diagram showing the distribution of KEGG genes in the genomes of *S. myotis* L7-1 and the four reference *Serratia* strains. **(B)** Venn diagram showing the distribution of COG genes in the genomes of *S.myotis* L7-1 and the four reference *Serratia* strains. www.ehbio.com/test/venn. **(C)** Pathway map of the type IV secretion system in *Serratia* L7-1 strain.

*Serratia* sp. are ubiquitous to different environments and show highly competitive fitness in versatile niches (Hover et al., 2016). The available genomic data of symbiotic fish bacteria is limited. To decipher the highly adaptive features of *S. myotis*, we next compared *S. myotis* L7-1 genome with four closely related bacterial genomes, including *Serratia quinivorans* 13,188 isolated from soil (Ashelford et al., 2003), *Serratia grimesii* BXF1 isolated from the pinewood nematodes (Nascimento et al., 2018), *Serratia liquefaciens* ATCC 27592 isolated from the spacecraft assembly facilities (Schuerger et al., 2013), and *Serratia proteamaculans* 336x isolated from the rinds of cheeses (Zhang et al., 2018). A total of 118 BGCs genes and 109 CAZyme genes were common to the five *Serratia* genomes (Supplementary Figure S5E). The unique gene clusters associated with SMs production in *S. myotis* L7-1 included betalactone, RiPP-like, arylpolyene, and siderophore. The unique CAZymes included glycosyl transferases and polysaccharide lyases (Supplementary Figure S5F). A total of 2,479 KEGG genes and 2,087 COG genes were common to the five *Serratia* genomes (Figures 4A,B). These unique genes in *S. myotis* L7-1 include those encoded for trafficking proteins, membrane fusion proteins, intracellular multiplication proteins, transport proteins, multiple enzymes and the others (Supplementary Tables S4, S5). Moreover, there was one notable feature that attracted our attention, the complete type IV secretion system with the particular plasmids.

L7-1 contained one chromosome and four plasmids, while other four reference bacterial genomes harbored one chromosome and no plasmids (Supplementary Figure S5). *S. myotis* L7-1 and all of the reference *Serratia* strains contained multiple secretion system genes, but only *S. myotis* L7-1 possessed a complete type IV secretion system (T4SS) located on the plasmids (Figure 4C). Many bacterial species deploy T4SSs to inject toxic effectors into target bacteria, thus inducing the death of rival cells (Rambow-Larsen and Weiss, 2004; Kutter et al., 2008; Juhas et al., 2010; Arslan-Aydogdu and Kimiran, 2018; Sgro et al., 2019). The type IV secretion system in *S. myotis* L7-1 included mainly type IV secretion system protein genes (virB1, 2, 4, 5, 6, 8, 9, 10, 11 and virD4) and a series of intracellular multiplication protein genes (icmB, E, G, J, K, L, O, P, T; Supplementary Table S4).

### Purification and characterization of bioactive compounds *via* the bioactivity-guided fractionation

**Figure 5:**
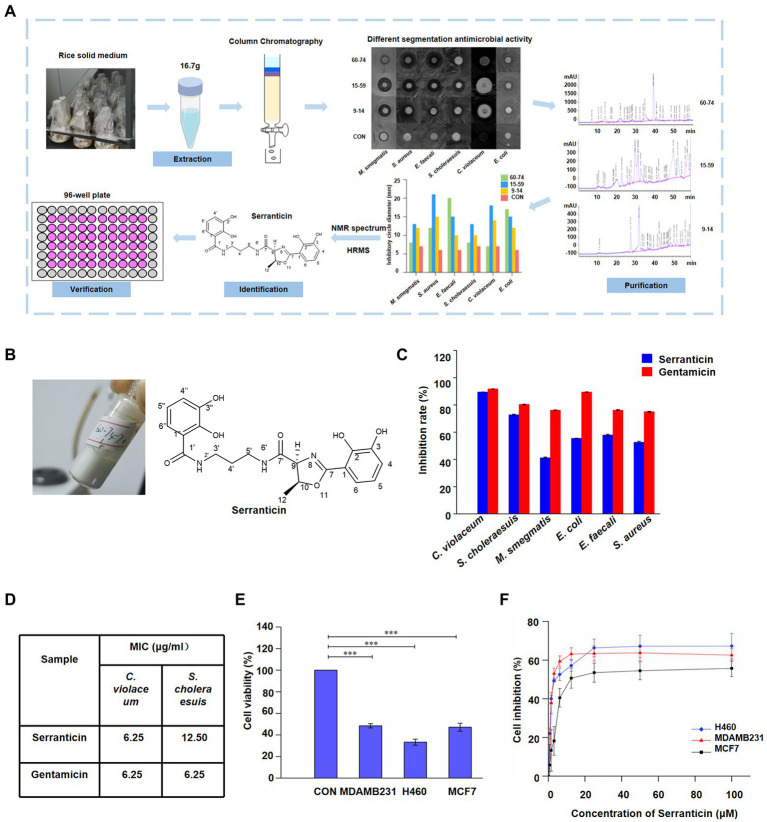
Purification and characterization of bioactive compound, serranticin. **(A)**, Workflow of the bioactivity-guided fractionation. **(B)**, Serranticin produced by *Serratia* L7-1 strain. ChemDraw 19.0 **(C)** *In-vitro* antimicrobial activity of the serranticin. Dates are the mean ± s.d. The experiments were repeated tree times with similar results. *Blue squares* serranticin, *rad squares* Gentamicin. **(D)** The MIC values for inhibition of pathogenic bacteria of serranticin and the standard Gentamicin. Origin 2022. **(E)**, *In-vitro* cytotoxicity activity of the serranticin. Dates are the mean ± s.d. The experiments were repeated tree times with similar results. Statistical significance of the test cell viability compared with the control group was determined using a two-tailed Student’s t-test; the *p* values were as follows:<0.0001 MDAMB231, H460, and MCF7 cells. **(F)**, Inhibition of cell proliferation by serranticin against H460, MDAMB231 and MCF7 cells. Dates are the mean ± s.d. The experiments were repeated tree times with similar results. *Blue diamond* H460, *rad triangle* MDAMB231, *black squares* MCF7. Origin 2022.

The versatile biosynthetic gene clusters and preliminary antimicrobial activity of the crude extract showed the great bioactive potential in *S. myotis* L7-1. To further determine the competitive roles of natural products, the bioactivity-guided fractionation assay against various pathogenic bacteria was conducted (Figure 5A). In total, 16.7 g of crude extract was obtained from a large-scale culture in rice solid medium. Bioactivity-guided purification and isolation strategy led to the isolation of bioactive fraction, compound 1 (61 mg; Figure 5B). It is proved that compound 1 displayed significant anti-bacteria activity as a leading product, compared with that of the other compounds from *S. myotis* L7-1. The results demonstrated that bioactivity-guided fractionation was an attractive and effective approach for profiling and screening of bioactive compounds.

Compound 1 was isolated as a white powder with a UV absorption at 210 nm (Figure 5B) and a specific optical rotation value of [α]_D_^20^–26.40^o^ (*c* 1.05, acetone; Supplementary Table S6). Its molecular formula is C_21_H_23_N_3_O_7_ (429.15). Its ^1^H NMR spectrum (600 MHz, in DMSO-*d*_6_, Supplementary Figure S7A) showed the following signals: *δ*_H_ 2.23 (3H, d, *J* = 6.4 Hz, H-12), 3.28 (2H, m, H-4′), 3.97 (2H, m, H-5′), 4.08 (2H, m, H-3′), 5.24 (1H, d, *J* = 7.4 Hz, H-9), 5.64 (1H, m, H-10), 7.45 (1H, t, *J* = 8.0 Hz, H-5″), 7.52 (1H, t, *J* = 7.9 Hz, H-5), 7.68 (1H, dd, *J* = 7.9, 1.4 Hz, H-4), 7.75 (1H, dd, *J* = 7.9, 1.4 Hz, H-4″), 7.85 (1H, dd, *J* = 7.9, 1.4 Hz, H-6), 8.04 (1H, dd, *J* = 8.0, 1.4 Hz, H-6″), 9.09 (1H, t, *J* = 6.0, NH-2′), 9.58 (1H, t, *J* = 5.9 Hz, NH-6′), 10.00 (2H, s, OH-3, 3′), 12.52 (s, OH-2) and 13.54 (s, OH-2′). The ^13^C NMR spectrum (150 MHz, in DMSO-*d*_6_, Supplementary Figure S7B) showed 21 signals: *δ*_C_ 20.83 (q, C-12), 29.08 (t, C-4′), 36.74 (t, C-5′), 36.85 (t, C-3′), 73.88 (d, C-9), 78.97 (d, C-10), 110.49 (s, C-1), 115.09 (s, C-1″), 117.21 (d, C-6″), 118.01 (d, C-4), 118.08 (d, C-5″), 118.82 (d, C-5), 118.89 (d, C-4″), 119.58 (d, C-6), 145.91 (s, C-3), 146.42 (s, C-3″), 148.45 (s, C-2), 149.92 (s, C-2″), 165.85 (s, C-7), 169.75 (s, C-1′), and 169.94 (s, C-7′). Based on its ^1^H and ^13^C NMR data, HRESIMS data, specific optical rotation value, and comparison with previous literature data (Kuo et al., 2011, 2012), compound 1 was proven to be identical to serranticin. The ^1^H and ^13^C NMR data of serranticin (1) were also summarized in Supplementary Table S7.

### Serranticin shows significant antimicrobial activity and cytotoxicity

To further determine the bioactivity of serranticin in pharmaceutical potential, human pathogenic bacteria and tumor cells were chosen to conduct the antibiotic assay. Serranticin was tested against several bacterial strains, including *C. violaceum*, *S. choleraesuis*, *M. smegmatis*, *E. coli*, *E. faecalis*, and *S. aureus*. Gentamicin (an antibiotic against both Gram-positive and negative bacteria) was used as the positive control. The results showed that serranticin exhibited strong antibacterial activity against both *C. violaceum* and *S. choleraesuis* at 50 μg/ml, with inhibition rate of 89.5 and 72.8%, respectively (Figure 5C). In addition, serranticin also displayed moderate activity toward *M. smegmatis*, *E*. *coli*, *E. faecalis*, and *S. aureus* with inhibition rate of 41.2%, 55.45%, 57.9% and 52.6%, respectively. The antimicrobial activity was studied further to evaluate the minimum inhibitory concentration (MIC) against *C. violaceum* and *S. choleraesuis* by broth microdilution method. Serranticin showed excellent antimicrobial activities against *C. violaceum* and *S. choleraesuis* with MIC values of 6.25 and 12.50 μg/ml, while the MIC value of gentamicin were 6.25 and 6.25 μg/ml (Figure 5D). Serranticin approximately rivaled the antimicrobial activity of the positive control gentamicin.

Antitumor activities of serranticin were tested by the MTT method using the human breast carcinoma MDA-MB-231, MCF7 and human lung cancer NCI-H460 cell lines. Serranticin inhibited the growth of three tested cell lines significantly and the cell viability values at 100 μM were given below, 48.5% (MDA-MB-231), 33.4% (NCI-H460), and 47.2% (MCF7; Figure 5E), respectively. The half-inhibitory concentration (IC50) of serranticin on MDA-MB-231, NCI-H460 and MCF7 cell lines were determined to be 2.8, 3.8 and 12.1 μM (Figure 5F), respectively. The data indicated that serranticin showed strong inhibitory activity against human cancer cell lines, suggesting that it can be a potential candidate for pharmaceutical application.

### *Serratia myotis* L7-1 confers protection for the colonization *via* metabolism

**Figure 6:**
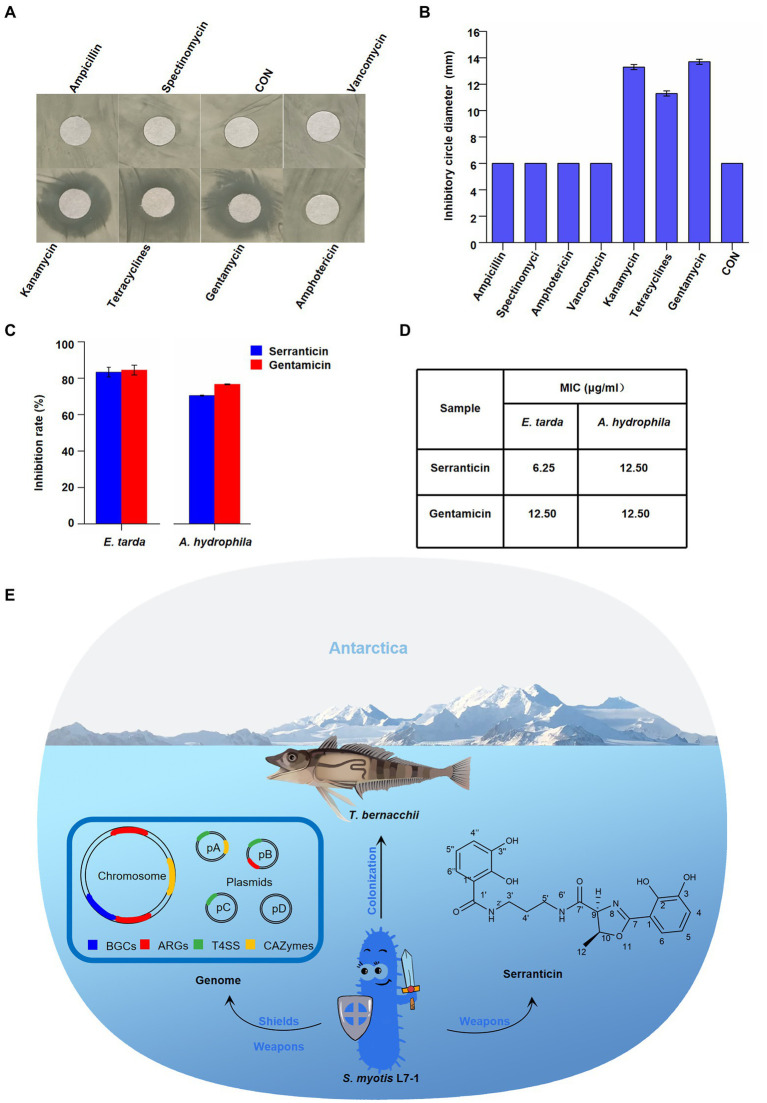
Effect of serranticin on bacteria antagonism. **(A)** Zone of inhibition of *S. myotis* L7-1 by antibiotics. **(B)** Antibiotic resistance activity of *S. myotis* L7-1. The diameter of circular filter paper sheets was 6 mm. Dates are the mean ± s.d. The experiments were repeated tree times with similar results. Origin 2022 **(C)**, *In-vitro* antimicrobial activity of the serranticin. Dates are the mean ± s.d. The experiments were repeated tree times with similar results. *Blue squares* serranticin, *rad squares* Gentamicin. Origin 2022. **(D)** The MIC values for inhibition of aquatic pathogenic bacteria of serranticin and the standard Gentamicin. Origin 2022. **(E)** Diagram illustrating how *S. myotis* L7-1 from *T. bernacchii* helps hosts adapt to the extreme Antarctic environment and resist pathogenic bacteria through the unique genomic features and production of serranticin.

It seems that *S. myotis* L7-1 harbored the ARGs to resist several antibiotics. To further determine the multiple resistance of *S. myotis* L7-1, the drug sensitivity test was conducted. Using the Kirby-Bauerdisca (K-B) agar diffusion method (Figure 6A), we tested the drug susceptibility with common clinical antibiotics at 1 mg/ml, including ampicillin, spectinomycin, vancomycin, kanamycin, tetracyclines, gentamycin and amphotericin. LB medium was used as negative control. The results indicated that the activities of ampicillin, spectinomycin, amphotericin and vancomycin were equivalent to the negative control for *S. myotis* L7-1 in terms of inhibition, with no significant zone of inhibition shown (Figure 6B). The diameters of the inhibition circle were 13.3, 11.3 and 13.7 cm for kanamycin, tetracyclines and gentamycin. From these results, it is clear that *S. myotis* can tolerate ampicillin, spectinomycin, amphotericin and vancomycin, while it is sensitive to kanamycin, tetracyclines and gentamycin.

The BGCs analyses of *S. myotis* reveal the potential production of bioactive compounds. The preliminary antagonism assays indicated that *S. myotis* L7-1 inhibits the growth of bacteria pathogens (Figure 2F). Next, the bioactivity-guided fractionation against aquatic pathogen was conduct, presenting the excellent active agent, serranticin. The antimicrobial activity of serranticin was determined *in vitro* for against *Edwardsiella tarda* and *Aeromonas hydrophila*, the serious bacteria pathogens of fish. The results showed that serranticin was active against *E. tarda* and *A. hydrophila* at 50 μg/ml, with inhibition rate of 83.3 and 70.5% (Figure 6C). The MIC values of the serranticin were determined by the broth microdilution method. The results indicated that serranticin had strong anti-aquatic pathogenic bacteria activity in inhibiting the growth of *E. tarda* (MIC: 6.25 μg/ml), and *A. hydrophila* (MIC: 6.25 μg/ml; Figure 6D). Positive control gentamicin had anti-bacteria activity with an MIC value of 12.5 μg/ml against *E. tarda* and 12.5 μg/ml against *A. hydrophila*. Serranticin displayed even stronger antimicrobial activity than positive control gentamicin, that contributes to the development of probiotics and potential utility in fishery industry.

## Discussion

The symbiotic microbiota have evolved environmental adaptation, and possess the potential production of abundant and novel natural products (Dou and Dong, 2019). However, there are still some issues that need to be resolved. For example, one overriding problem is how to find the core microbe to play the key roles in the environmental adaptation and the potential to be the industrial strains (Macintyre et al., 2014). Here, we showed the methods to discover the valuable strains from the huge nature resources, recruiting cultivable method and the genomic analyses (TB-S, TB-I). In our study, 58 cultivable bacteria have been isolated. The isolated strains from such extreme environment would offer excellent model organism to investigate the physiology of the symbionts and understand their ecological role in the host. However, it was still challenging to select the representative strain. Through the comparison of populations of TB-S and TB-I, *S. myotis* became the potential target genus among the isolated symbiotic bacteria as the prevalent genus with the high adaptability and bioactive potential. The intrinsic existence of *S. myotis* was also proved by the meta-genomic analyses of fish sample (data not shown). Although more research is needed, it seems to be an effective way to pick up the potential bacteria.

*Serratia* bacteria show versatile niche occupation abilities in plant, soil, water and animals, associated with a wide range of different hosts (Kalbe et al., 1996; Kamensky et al., 2003; Tsaplina et al., 2015; Zheng et al., 2021). It is still unclear what’s the underlying causes in environmental adaptation of *Serratia* sp. (Nascimento et al., 2018). In our study, the general genome features of *S. myotis* associated with the adaptation of extreme environment were characterized, like the genes related to cold adaptation (heat shock protein) and stress response. Furthermore, we speculate that the reservoir of antibiotic-resistance potential and multiple antibiotic resistance mechanisms facilitated *S. myotis* to survive in host under the selection pressure in adverse environment. Here, most of the ARGs located on the chromosome confers the intrinsic resistance of *S. myotis*, which is different from the other plasmid-carrying bacteria in an antibiotic-influenced environment (Luisa et al., 2018). However, most research on antibiotic-resistant bacteria (ARB) have focused on the mainland and offshore oceans, and its extent in polar regions has seldom been investigated (Na et al., 2021). It seems that it has been underestimated that Antarctic environment provides a natural reservoir for antibiotic resistance genes. Furthermore, the existence of type VI transporter system in plasmid of *S. myotis* was not found in the other four *Serratia* sp. T4SSs are used for the delivery of bacterial effector proteins across the membrane of eukaryotic target cells, contributing directly to the bacterial pathogenicity (Aguilar et al., 2010). The type IV secretion system may contribute to the evolvement of *S. myotis* as the toxin transporters in inter-bacterial antagonism and communication. Although more functional studies need to be tested, our genomic characterization could be a useful step in addressing the adaptation of symbionts in Antarctic fish in future.

*Serratia* bacteria show biosynthesized ability of a broad range of secondary metabolites (SMs; Soenens and Imperial, 2019). The ecological function of *Serratia* has been studied with multi-species by metabolome analyses (Clements et al., 2019). However, it remains open if SMs of *Serratia* from the Antarctic fish are involved in novel function. Our BGCs analyses indicated that *S. myotis* has the ability of abundant bioactive compounds production. We showed the purification and characterization of serranticin from *S. myotis*. The critical roles of antitumor activity and antibacterial activity, make serranticin tailored for the pharmaceutic and bio-control application. The structure of serranticin is analogous to siderophores, which are iron chelators in bacterial iron acquisition (Page, 2019). It appears that serranticin has never been reported in *S. myotis* in previous reports (Kuo et al., 2011). Compared with the sequence of biosynthetic cluster in the published data (Seyedsayamdost et al., 2012), two serranticin clusters of 21 kb and 12.3 kb, were assigned as src cluster in *S. myotis* (Supplementary Figure S8; Supplementary Tables S8, S9). The high homologous cluster sequence and the accepted role of this compound as siderophore give the hints of interpreting the role of serranticin in bacterial competition. Furthermore, serranticin inhibited the growth of *Aeromonas hydrophila* and *Edwardsiella piscicida*, which are harmful to the fish health. These results indicate that *Serratia* outcompete some fish pathogens. It is reasonable to suspect then that *S. myotis* can benefit from the production of serranticin and may even facilitate the host health. The obtained results led us to propose a hypothetical model. The strong activity in bacteria antagonism determines its ecological role as an active defense system in direct interactions with symbiotic microorganisms, while the antibiotic-resistance acts as a passive defense system in extreme environment (Figure 6E).

Our work emphasizes the discovery and importance of a symbiotic bacteria of fish from Antarctic, *S. myotis* L7-1. Host adaptability mechanism and its biosynthetic potential were investigated using the whole genomic sequencing and chemical characterization. Our study provides the first insights into the adaptative role of symbiotic microbiota in Antarctic fish from the extreme environment, that could pave the way for future research. Furthermore, Antarctic bacteria are hardly studied as potential sources of novel natural products for exploitation in medicine, agriculture, and food. Further detailed study on bioactive compounds could shed light on the discovery of novel natural products from the symbiotic microbiomes of fish.

### Statistics and reproducibility

Unless specifically noted, each experiment was repeated three or more times independently. Data were collected from three biological and three technical replicates, unless otherwise noted. Data shown in column graphs or line chart represent mean ± the standard deviation (SD), as indicated in the figure legends. Statistical analysis was performed with Origin 2022.^1^ Graph preparation was performed,^2^ itol.embl.de and ChemDraw 19.0. More details were given in the figure legends and methods.

## Data availability statement

The data presented in the study are deposited in the National Center for Biotechnology Information (NCBI, https://www.ncbi.nlm.nih.gov/) repository. The entire 16sRNA gene sequence dataset in this paper has been uploaded in the NCBI Sequence Read Archive (accession no. PRJNA852421). The whole-genome and the sequences of 4 plasmids have been deposited at the NCBI genome database under the accession numbers CP099707 (Chromosome), CP099708 (pL71A), CP099709 (pL71B), CP099710 (pL71C), and CP099711 (pL71D).

## Author contributions

YX performed most of the experiments and analyzed the results. FY and YC participated in isolation of strains. JD and RL contributed to the genomic profiling assay. WZ collected the samples. ZZ analyzed the data about structure characterization. JF reviewed the manuscript. LC conceived the idea and supervised the project. XY designed, coordinated the study, provided all the infrastructure, supervised the project, and wrote the manuscript. All authors contributed to the article and approved the submitted version.

## Funding

This work was supported by grants from National Natural Science Foundation of China 42006086 and 91951210, Shanghai sailing program 20YF1416900, Science and Technology Commission of Shanghai Municipality STCSM 20050501700, the National Key Research and Development Program of China (2018YFD0900601).

## Conflict of interest

The authors declare that the research was conducted in the absence of any commercial or financial relationships that could be construed as a potential conflict of interest.

## Publisher’s note

All claims expressed in this article are solely those of the authors and do not necessarily represent those of their affiliated organizations, or those of the publisher, the editors and the reviewers. Any product that may be evaluated in this article, or claim that may be made by its manufacturer, is not guaranteed or endorsed by the publisher.

## Footnotes

^1^ www.ehbio.com/test/venn

^2^ www.figdraw.com
