# Supplementary material for: A symbiotic bacterium of Antarctic fish reveals environmental adaptability mechanisms and biosynthetic potential towards antibacterial and cytotoxic activities
Source: Front Microbiol. 2023 Jan 13;13:1085063. doi: 10.3389/fmicb.2022.1085063 (PMC9882997; doi:10.3389/fmicb.2022.1085063)
Supplement: Supplementary file 1 [file Data_Sheet_1.docx]

**A symbiotic bacterium of Antarctic fish reveals** **environmental adaptability mechanisms and biosynthetic potential towards antibacterial and cytotoxic activities**

Yu Xiao^1^, Fangfang Yan^1^, Yukun Cui^1^, Jiangtao Du^1^, Guangzhao Hu^1^, Wanying Zhai^2^, Rulong Liu^1^, Zhizhen Zhang^3^, Jiasong Fang^1^, Liangbiao Chen^2*^ and Xi Yu^1*^

**running head:** Antarctic bacteria genomic and metabolites

**Address**: ^1^Shanghai Engineering Research Center of Hadal Science and Technology, College of Marine Sciences, Shanghai Ocean University, Shanghai, 201306, China

^2^ International Research Center for Marine Biosciences, Ministry of Science and Technology, Shanghai Ocean University, Shanghai, 201306, China

^3^Ocean College, Zhoushan Campus, Zhejiang University, Zhoushan, 316021, China

*corresponding author: XY (x[yu@shou.edu.cn](mailto:yu@shou.edu.cn), +8615332036650);

LC ([lbchen@shou.edu.cn](mailto:lbchen@shou.edu.cn), +8615618063251).

**Key words:** Antarctic fish, symbiotic bacteria, *Serratia*, genome, bioactive metabolites

**Figure S1**


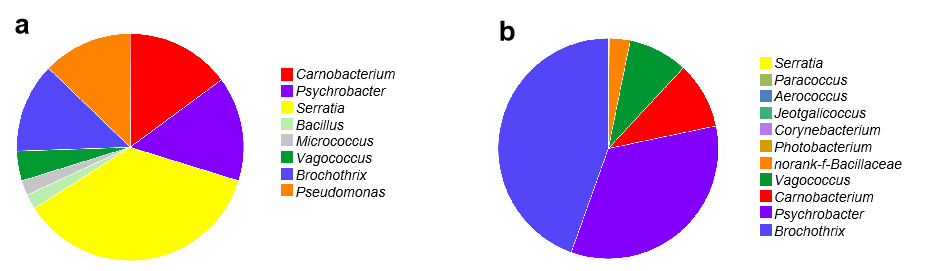


**Supplementary Figure S1 Identification of fish symbiotic bacteria. a,** Composition and relative abundance of symbiotic bacteria of TB-I. *Serratia* (36.2%) (*Serratia myotis*)，*Carnobacterium* (14.9%) (*Carnobacterium maltaromaticum*, *Carnobacterium divergens*, *Carnobacterium Mobile*, *Carnobacterium funditum*, *Carnobacterium antarcticm*), *Psychrobacter* (14.9%) (*Psychrobacter maritimus*), *Pseudomonas* (12.8%) (*Pseudomonas paracarnis*, *Pseudomonas fildesensis*, *Pseudomonas kitaguniensis*), *Brochothrix* (12.8%) (*Brochothrix themosphacta*), *Vagococcus* (4.3%) (*Vagococcus salmoninarum*), *Micrococcus* (2.1%) (*Micrococcus luteus*) and *Bacillus* (2.1%) (*Bacillus proteolyticus*). **b,** Composition and relative abundance of symbiotic bacteria of TB-S. *Brochothrix* (44.5%), *Psychrobacter* (33.8%), *Carnobacterium* (9.8%), *Vagococcus* (8.7%), *norank-f-Bacillaceae* (3.1%), *Photobacterium* (0.04%), *Corynebacterium* (0.03%), *Jeotgalicoccus* (0.02%), *Aerococcus* (0.02%), *Serratia* (0.02%) and *Paracoccus* (0.003%) Origin 2022.

**
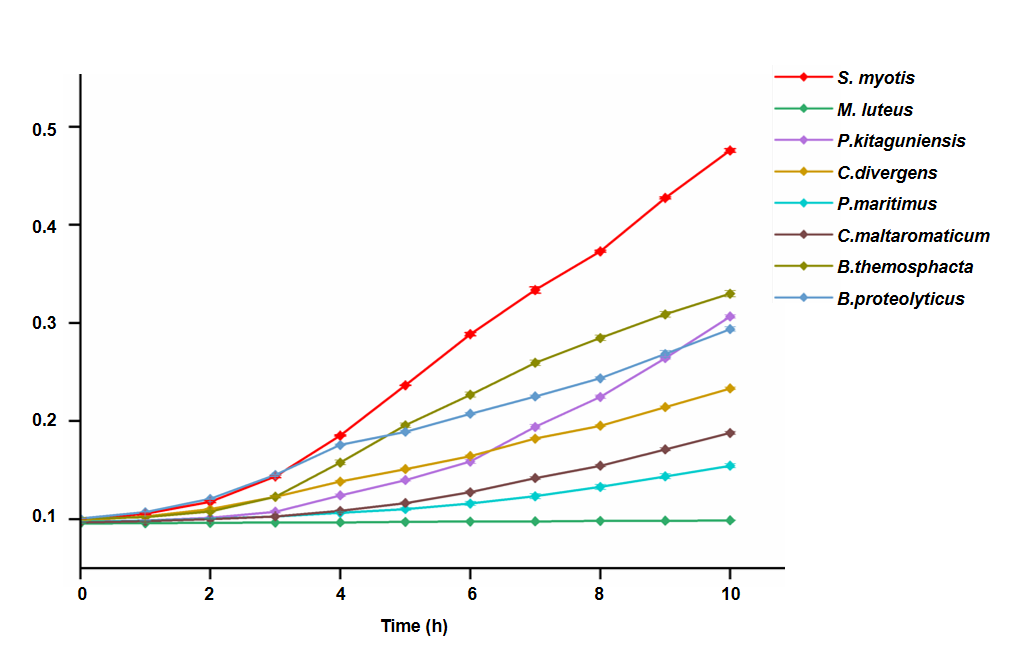
Figure S2**

**Supplementary Figure S2 Growth curves of nine symbiotic bacteria from Antarctic fish under minimal nutrient media.** Nine symbiotic bacteria: *Serratia myotis*, *Micrococcus luteus*, *Pseudomonas kitaguniensis*, *Carnobacterium divergens*, *Psychrobacter maritimus*, *Carnobacterium maltaromaticum*, *Brochothrix themosphacta*, *Bacillus proteolyticus*. Origin 2022


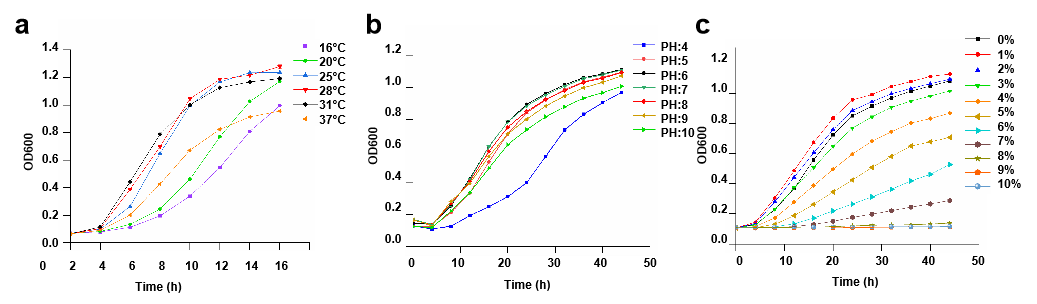
**Figure S3**

**Supplementary Figure S3 Growth curves of *S. myotis* L7-1 under different growth conditions.** **a,** Different temperature (16℃, 20℃, 25℃, 28℃, 31℃, 37℃). **b,** Different pH values (4-10, interval 1). **c,** Different salinity (w/v (0-10%, interval 1%). Three parallel groups were set up for each group. Origin 2022

**Figure S4**


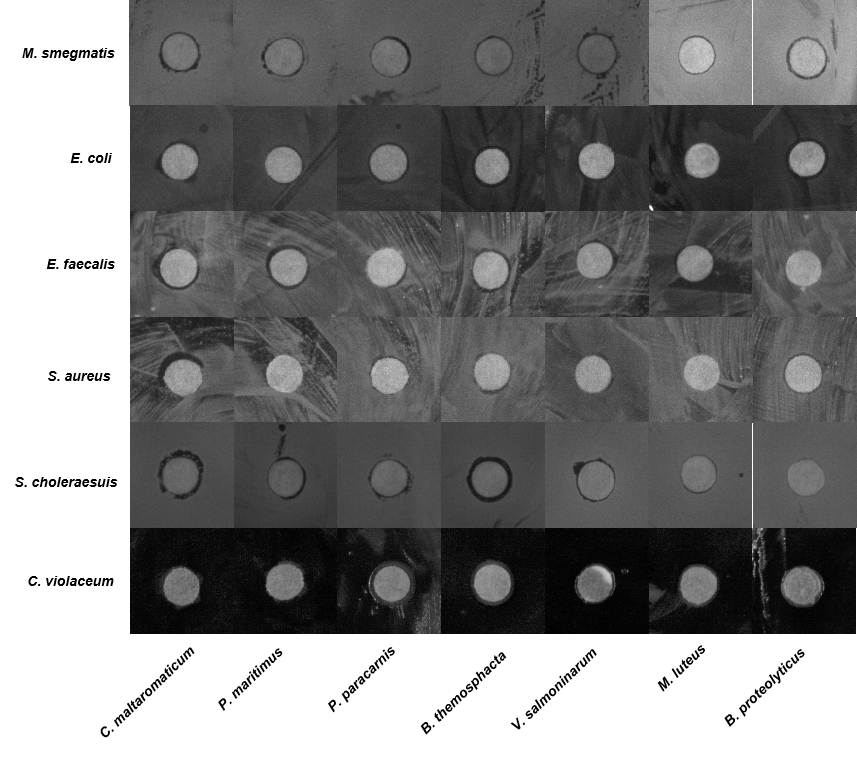


**Supplementary Figure S4 The antibacterial activity of secondary metabolites of symbiotic bacteria.** Zone of inhibition of the metabolites produced by seven symbiotic bacteria isolated from *T. bernacchii.*


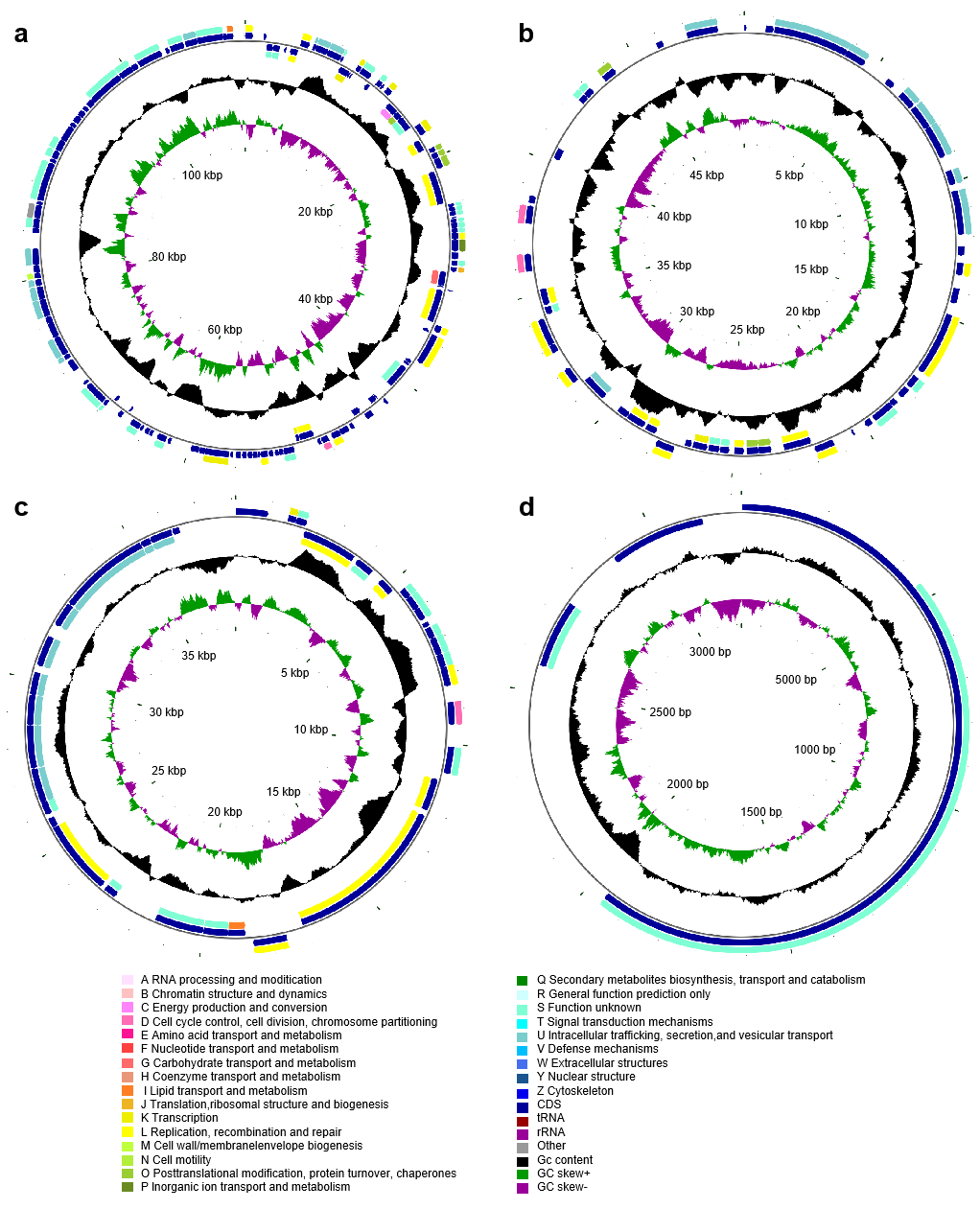
**Figure S5**


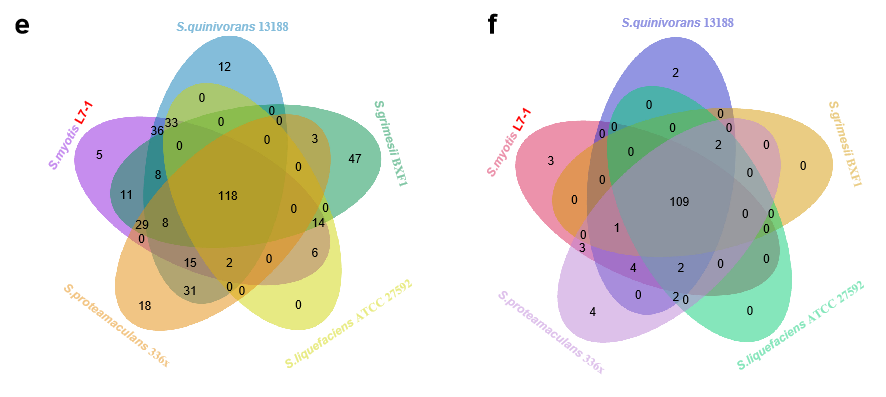


**Supplementary Figure S5 Comparative genomics indicates the specific adaptability mechanisms.** Circular map of plasmid of *S. myotis* L7-1. **a,** Plasmid A. 110,419 bp (pA), with a GC content of 55.10%, and 125 genes with coding sequences. A total of 0 tRNA, 0 rRNA and 1 sRNA were detected. A total of 127 genes were predicted to have COG functional annotation. **b,** Plasmid B. 49,145 bp (pB), with a GC content of 44.83%, and 48 genes with coding sequences. A total of 0 tRNA, 0 rRNA and 2 sRNA were detected. A total of 50 genes were predicted to have COG functional annotation. **c,** Plasmid C. 38,106 (pC), with a GC content of 50.14%, and 36 genes with coding sequences. A total of 0 tRNA, 0 rRNA and 1 sRNA were detected. A total of 37 genes were predicted to have COG functional annotation. **d,** Plasmid D. 3,223 bp (pD), with a GC content of 56.28%, and 4 genes with coding sequences. A total of 0 tRNA, 0 rRNA and 1 sRNA were detected. A total of 4 genes were predicted to have COG functional annotation. From outside to inside Ring 1 and 4 depicts CDS in the positive strand and negative strand, different colors indicate different COG functional classifications. Ring 2 and 3 depicts CDS, tRNA and rRNA in the positive strand and negative strand. Ring 5 and 6 represents GC content and GC skew, respectively.cloud.majorbio.com **e,** Venn diagram showing the distribution of secondary metabolic synthesis gene cluster genes in the genomes of *S. myotis* L7-1 and the four reference *Serratia* strains. A total of 118 secondary metabolic synthesis gene cluster genes were common to the five *Serratia* genomes. The unique gene cluster gene in *S. myotis* L7-1 included betalactone, RiPP-like and arylpolyene, siderophore, associated with SM production. **f,** Venn diagram showing the distribution of CAZyme genes in the genomes of *S. myotis* L7-1 and the four reference *Serratia* strains. A total of 109 CAZyme genes were common to the five *Serratia* genomes. The unique CAZyme gene in *S. myotis* L7-1 included glycosyl transferases and polysaccharide lyases. www.ehbio.com/test/venn.

**Figure S6**


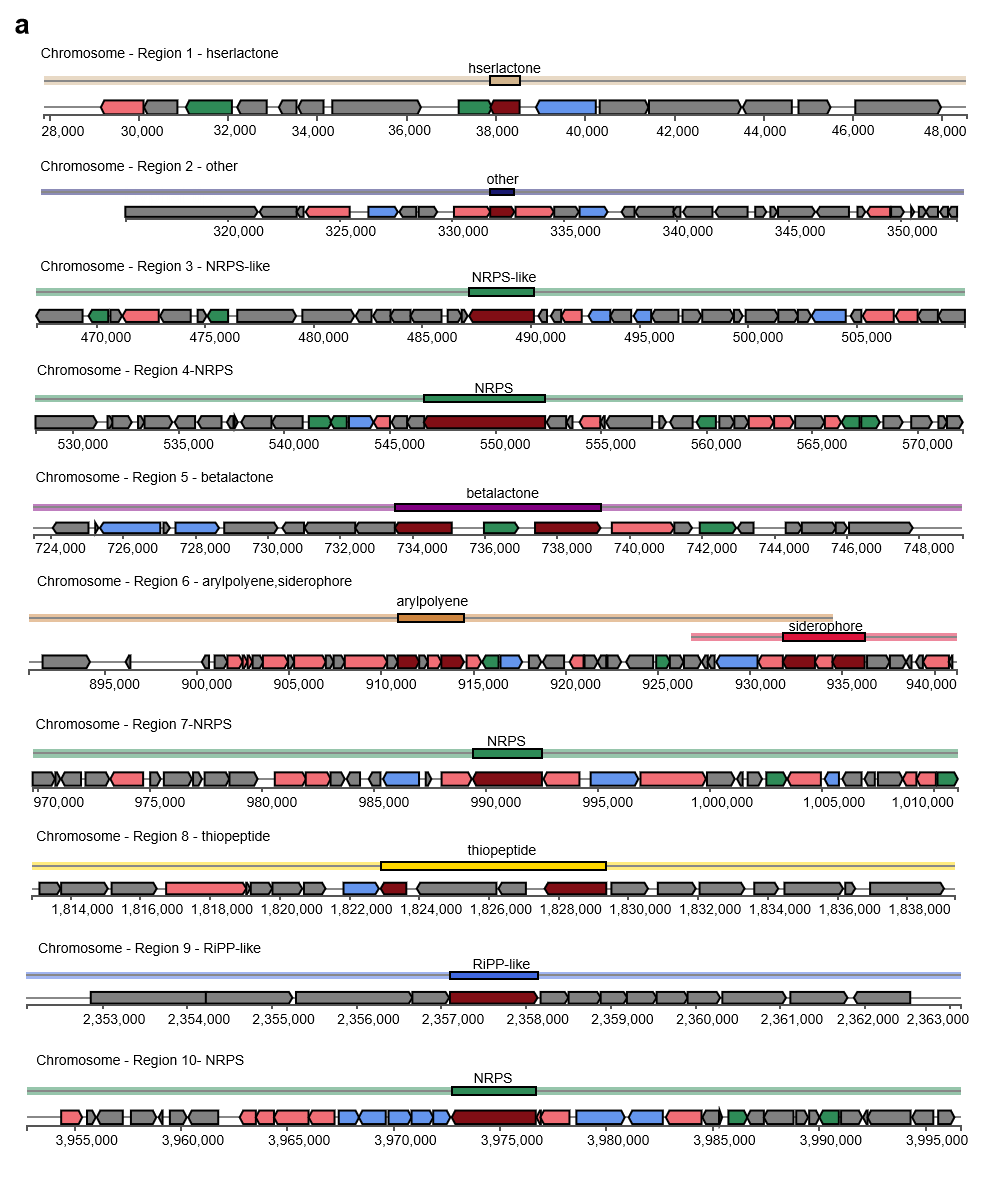


**Supplementary Figure S6** **Genomic annotation and BGCs prediction of *S. myotis* L7-1.** 10 secondary metabolic gene clusters of *S. myotis* L7-1. antismash. secondarymetabolites.org


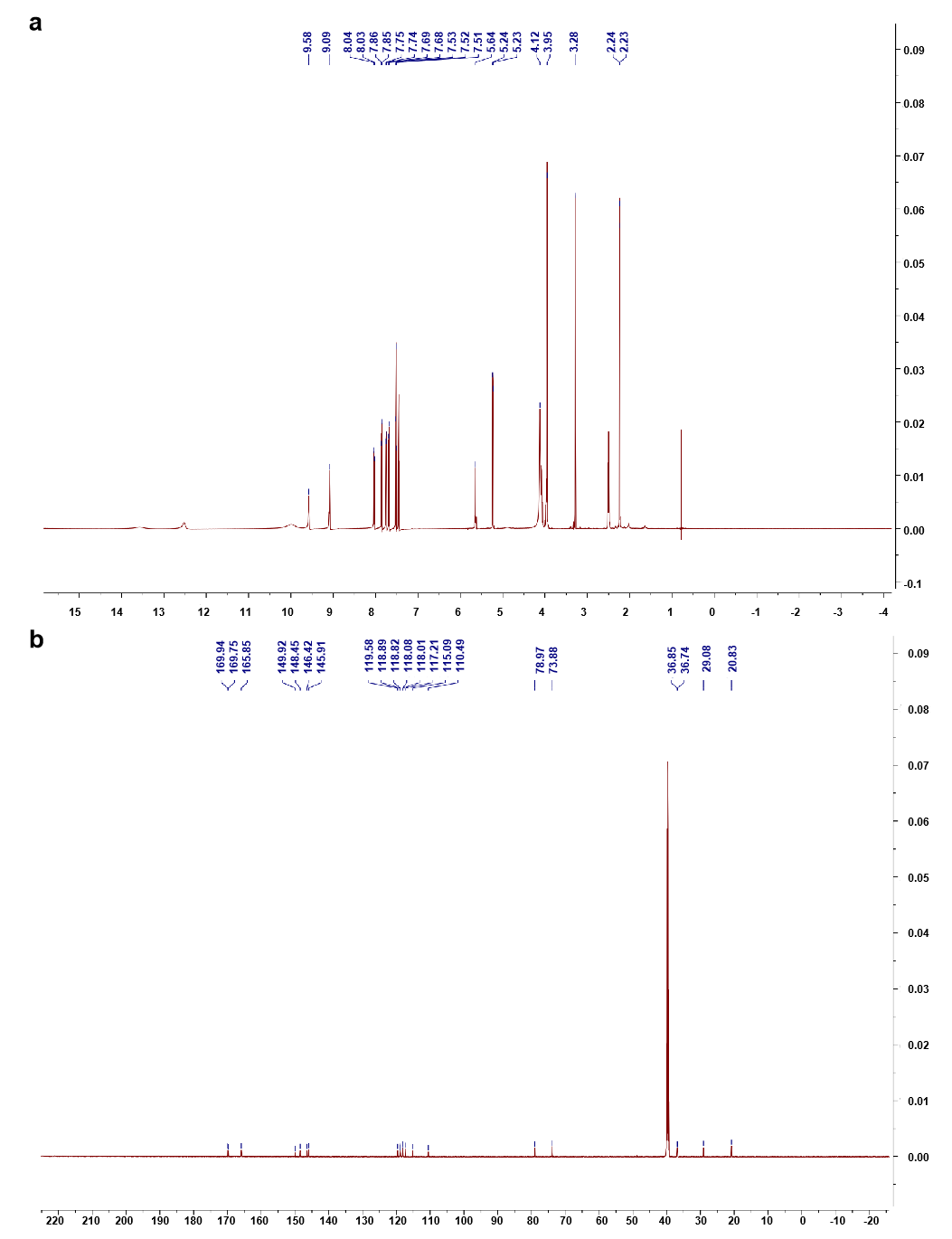
**Figure S7**

**Supplementary Figure S7 Identification of the bioactive compound serranticin.** **a,** ^1^H-NMR spectrum of srranticin in DMSO-*d*_6_. **b,** ^13^C-NMR spectrum of serranticin in DMSO-*d*_6_.

**Figure S8**

**
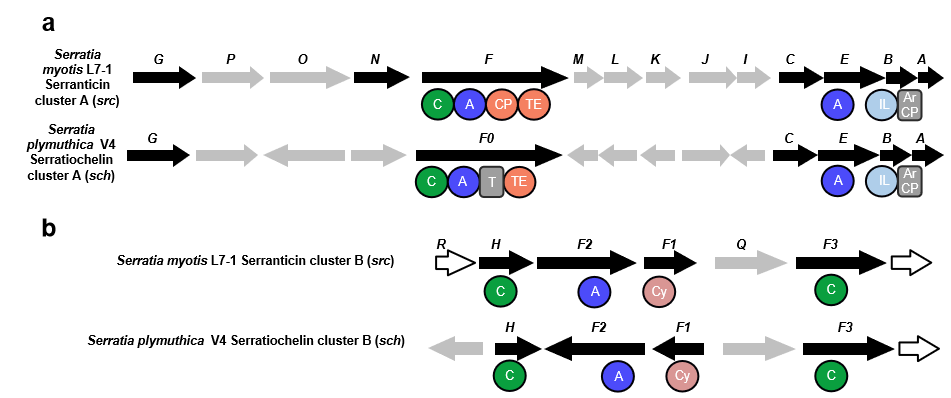
**

**Supplementary Figure S8 Serranticin gene clusters. a,** Comparison of serranticin cluster A with the *Serratia plymuthica* V4 serratiochelin cluster A. The sequences and syntenies of these two clusters are highly homologous. **b,** Comparison of serranticin cluster B with the *Serratia plymuthica* V4 serratiochelin cluster B. The sequences and syntenies of these two clusters are highly homologous.

**Supplementary Table S1 Isolation of fish symbiotic bacteria.** Separation medium formulation.

| Medium | Formulation | PH |
| --- | --- | --- |
| LB agar | yeast extract 5 g, NaCl 5 g, pepptone 10 g, agar 15 g | natural PH |
| NA | trypeptone 10 g, NaCl 5 g, beef extract 3 g, agar 15 g | natural PH |
| TSA | FeSO_4_.7H_2_O 0.01 g, MgSO_4_.7H_2_O 0.2 g, K_2_HPO_4_ 0.5 g, beef extract 2 g, yeast extract 2 g, ammonium acetate 3 g, peptone 6 g，glucose 40 g, agar 15 g | PH 6.5 |
| RCM | L-Cysteine hydrochloride 0.5 g, solublestarch 1 g, Sodium acetate 3 g, NaCl 3 g, yeast extract 3 g, glucose 5 g, beef extract 10 g, peptone 10 g, 15 g agar | PH 8.5 |
| MASM | KH_2_PO_4_ 0.1 g, NH_4_Cl 0.3 g, KCl 0.5 g, Trace element 1 mL, Vitamin mixture 1 mL, Vitamin B1 1 mL, Vitamin B12 1 mL, CaCl_2_.2H_2_O 1.4 g, N-acetylglucosamine 2 g, Na_2_SO_4_ 4 g, MgCl.6H_2_O 5 g, glucose 5.2 g, NaHCO_3_ 20 mM 20 mL, NaCl 26 g, agar 15 g | PH 7-8 |
| PDA | glucose 20 g, potato 200g, agar 15 g | natural PH |
| YMG | yeast extract 4 g, malt extract 10 g, glucose 20 g, agar 15 g | natural PH |
| MM | FeSO_4_.7H_2_O 0.01 g, MgSO_4_.7H_2_O 0.2 g, K_2_HPO_4_ 0.5 g, (NH_4_)_2_SO_4_ 1g, glucose 10 g, agar 15 g | natural PH |
| MN | Na_2_HPO_4_ 5.96g, K_2_HPO_4_ 3 g, NH_4_Cl 1 g, NaCl 0.5 g, MgSO_4_ 0.058 g, glucose 5 g | PH 7 |

**Supplementary Table S2 Identification of *Serratia* strains.** 17 strains of *S. myotis* *16S* rDNA similarity, interspecies similarity and isolation medium.

| Name | Top-hit taxon | Top-hit taxon | Isolation medium | Interspecific similarity (%) |
| --- | --- | --- | --- | --- |
| L7-1 | *S. myotis* | 99.71 | LB | 99.70-100 |
| E7 | *S. myotis* | 99.78 | LB | 99.70-100 |
| F7S | *S. myotis* | 99.57 | LB | 99.40-100 |
| F7Z1 | *S. myotis* | 99.57 | LB | 99.70-100 |
| FLS7 | *S. myotis* | 99.78 | LB | 99.70-100 |
| L7-2 | *S. myotis* | 99.50 | LB | 99.40-99.70 |
| LD7 | *S. myotis* | 99.71 | LB | 99.70-100 |
| LM7-1 | *S. myotis* | 99.64 | MASM | 99.70-100 |
| LM7-3 | *S. myotis* | 99.57 | MASM | 99.70-100 |
| M7-1- | *S. myotis* | 99.64 | MM | 99.70-100 |
| N7-1 | *S. myotis* | 99.71 | NA | 99.70-100 |
| P7 | *S. myotis* | 99.78 | PDA | 99.70-100 |
| PE7 | *S. myotis* | 99.86 | PDA | 99.70-100 |
| RC7-1 | *S. myotis* | 99.78 | RCM | 99.70-100 |
| RC7 | *S. myotis* | 99.78 | RCM | 99.70-100 |
| SE7 | *S. myotis* | 99.78 | TSA | 99.70-100 |
| TS7-1 | *S. myotis* | 99.71 | TSA | 99.70-100 |

**Supplementary Table S3** Composition and number of other antibiotic resistance genes of *S. myotis* L7-1.

| Antibiotics | N |
| --- | --- |
| acridine dye | 9 |
| aminoglycoside antibiotic | 32 |
| antibacterial free fatty acids | 2 |
| benzalkonium chloride | 1 |
| bicyclomycin | 1 |
| diaminopyrimidine antibiotic | 15 |
| elfamycin antibiotic | 1 |
| fosfomycin | 8 |
| glycopeptide antibiotic | 19 |
| glycylcycline | 17 |
| lincosamide antibiotic | 21 |
| monobactam | 24 |
| mupirocin | 2 |
| nitrofuran antibiotic | 2 |
| nitroimidazole antibiotic | 12 |
| nucleoside antibiotic | 4 |
| oxazolidinone antibiotic | 13 |
| penem | 26 |
| phenicol antibiotic | 52 |
| pleuromutilin antibiotic | 19 |
| rhodamine | 1 |
| rifamycin antibiotic | 27 |
| streptogramin antibiotic | 16 |
| sulfonamide antibiotic | 11 |
| triclosan | 18 |

**Supplementary Table S4** Unique KEGG genes of *S. myotis* L7-1 compared to the four reference strains (*S. quinivorans* 13188, *S. grimesii* BXF1, *S. liquefaciens* ATCC 27592, and *S. proteamaculans* 336x).

| KEGG | KO Description |
| --- | --- |
| K03366 | butA, budC; meso-butanediol dehydrogenase (S, S)-butanediol dehydrogenase / diacetyl reductase [EC:1.1.1.- 1.1.1.76 1.1.1.304] |
| K12203 | dotB, traJ; defect in organelle trafficking protein DotB [EC:7.2.4.8] |
| K12204 | dotC, traI; defect in organelle trafficking protein DotC |
| K12205 | dotD, traH; defect in organelle trafficking protein DotD |
| K05275 | E1.1.1.65; pyridoxine 4-dehydrogenase [EC:1.1.1.65] |
| K07026 | E3.1.3.70; mannosyl-3-phosphoglycerate phosphatase [EC:3.1.3.70] |
| K17204 | eryE; erythritol transport system ATP-binding protein |
| K11202 | fryB; fructose-like PTS system EIIB component [EC:2.7.1.-] |
| K11203 | fryC, frvB; fructose-like PTS system EIIC or EIIBC or EIIABC component |
| K06048 | gshA, ybdK; glutamate---cysteine ligase / carboxylate-amine ligase [EC:6.3.2.2 6.3.-.-] |
| K11004 | hlyB, cyaB; ATP-binding cassette, subfamily B, bacterial HlyB/CyaB |
| K11003 | hlyD, cyaD; membrane fusion protein, hemolysin D |
| K12206 | icmB, dotO; intracellular multiplication protein IcmB [EC:7.2.4.8] |
| K12209 | icmE, dotG; intracellular multiplication protein IcmE |
| K12211 | icmG, dotF; intracellular multiplication protein IcmG |
| K12212 | icmJ, dotN; intracellular multiplication protein IcmJ |
| K12213 | icmK, traN, dotH; intracellular multiplication protein IcmK |
| K12214 | icmL, traM, dotI; intracellular multiplication protein IcmL |
| K12217 | icmO, trbC, dotL; intracellular multiplication protein IcmO [EC:7.2.4.8] |
| K12218 | icmP, trbA; intracellular multiplication protein IcmP |
| K12222 | icmT, traK; intracellular multiplication protein IcmT |
| K01182 | IMA, malL; oligo-1,6-glucosidase [EC:3.2.1.10] |
| K07045 | K07045; uncharacterized protein |
| K07498 | K07498; putative transposase |
| K12227 | K12227, traL; TraL protein |
| K17202 | K17202, eryG; erythritol transport system substrate-binding protein |
| K17203 | K17203, eryF; erythritol transport system permease protein |
| K18991 | mtrA; AraC family transcriptional regulator, activator of mtrCDE |
| K01788 | nanE; N-acylglucosamine-6-phosphate 2-epimerase [EC:5.1.3.9] |
| K01174 | nuc; micrococcal nuclease [EC:3.1.31.1] |
| K18640 | parM, stbA; plasmid segregation protein ParM |
| K23991 | ptsA, fryA; multiphosphoryl transfer protein [EC:2.7.3.9 2.7.1.-] |
| K02856 | rhaT; L-rhamnose-H+ transport protein |
| K08172 | shiA; MFS transporter, MHS family, shikimate and dehydroshikimate transport protein |
| K03465 | thyX, thy1; thymidylate synthase (FAD) [EC:2.1.1.148] |
| K12228 | trbB; TrbB protein |
| K20531 | trbF; type IV secretion system protein TrbF |
| K20532 | trbG; type IV secretion system protein TrbG |
| K20533 | trbI; type IV secretion system protein TrbI |
| K03194 | virB1; type IV secretion system protein VirB1 |
| K03195 | virB10, lvhB10; type IV secretion system protein VirB10 |
| K03196 | virB11, lvhB11; type IV secretion system protein VirB11 [EC:7.4.2.8] |
| K03197 | virB2, lvhB2; type IV secretion system protein VirB2 |
| K03199 | virB4, lvhB4; type IV secretion system protein VirB4 [EC:7.4.2.8] |
| K03200 | virB5, lvhB5; type IV secretion system protein VirB5 |
| K03201 | virB6, lvhB6; type IV secretion system protein VirB6 |
| K03203 | virB8, lvhB8; type IV secretion system protein VirB8 |
| K03204 | virB9, lvhB9; type IV secretion system protein VirB9 |
| K03205 | virD4, lvhD4; type IV secretion system protein VirD4 [EC:7.4.2.8] |
| K16695 | wzxC; lipopolysaccharide exporter |
| K03829 | yedL; putative acetyltransferase [EC:2.3.1.-] |

**Supplementary Table S5** Unique COG genes of *S. myotis* L7-1 compared to the four reference strains (*S. quinivorans* 13188, *S. grimesii* BXF1, *S. liquefaciens* ATCC 27592, and *S. proteamaculans* 336x).

| COG | COG Description |
| --- | --- |
| COG3253 | Coproheme decarboxylase/chlorite dismutase |
| COG3415 | CRISPR-associated protein Csa3, CARF domain |
| COG2390 | DNA-binding transcriptional regulator LsrR, DeoR family |
| COG1479 | DNAse/DNA nickase specific for phosphorothioated or glycosylated phage DNA, GmrSD/DndB/SspE family, contains DUF262 and HNH nuclease domains |
| COG1525 | Endonuclease YncB, thermonuclease family |
| COG5039 | Exopolysaccharide biosynthesis protein EpsI, predicted pyruvyl transferase |
| COG3401 | Fibronectin type 3 domain |
| COG2170 | Gamma-glutamyl:cysteine ligase YbdK, ATP-grasp superfamily |
| COG3306 | Glycosyltransferase involved in LPS biosynthesis, GR25 family |
| COG3385 | IS4 transposase InsG |
| COG3769 | Mannosyl-3-phosphoglycerate phosphatase YedP/MpgP, HAD superfamily |
| COG5520 | O-Glycosyl hydrolase |
| COG3409 | Peptidoglycan-binding (PGRP) domain of peptidoglycan hydrolases |
| COG4938 | Predicted ATPase |
| COG4889 | Predicted helicase |
| COG5618 | Predicted periplasmic lipoprotein |
| COG3010 | Putative N-acetylmannosamine-6-phosphate epimerase |
| COG0535 | Radical SAM superfamily maturase, SkfB/NifB/PqqE family |
| COG1715 | Restriction endonuclease Mrr |
| COG3587 | Restriction endonuclease, type III RM system |
| COG1351 | Thymidylate synthase ThyX, FAD-dependent family |
| COG3958 | Transketolase, C-terminal subunit |
| COG3959 | Transketolase, N-terminal subunit |
| COG3547 | Transposase |
| COG4753 | Two-component response regulator, YesN/AraC family, consists of REC and AraC-type DNA-binding domains |
| COG3170 | Type IV pilus assembly protein FimV |
| COG0630 | Type IV secretory pathway ATPase VirB11/Archaellum biosynthesis ATPase ArlI/FlaI |
| COG3736 | Type IV secretory pathway, component VirB8 |
| COG3701 | Type IV secretory pathway, TrbF component |
| COG2948 | Type IV secretory pathway, VirB10 component |
| COG3838 | Type IV secretory pathway, VirB2 component (pilin) |
| COG3702 | Type IV secretory pathway, VirB3 component |
| COG3451 | Type IV secretory pathway, VirB4 component |
| COG3704 | Type IV secretory pathway, VirB6 component |
| COG3504 | Type IV secretory pathway, VirB9 components |
| COG3843 | Type IV secretory pathway, VirD2 component (relaxase) |
| COG3505 | Type IV secretory pathway, VirD4 component, TraG/TraD family ATPase |
| COG4925 | Uncharacterized conserved protein |
| COG4249 | Uncharacterized conserved protein, contains caspase domain |
| COG5470 | Uncharacterized conserved protein, DUF1330 family |
| COG5639 | Uncharacterized conserved protein, DUF2274 domain |
| COG4541 | Uncharacterized membrane protein |
| COG4694 | Wobble nucleotide-excising tRNase |

**Supplementary Table S6 Identification of the bioactive compound serranticin.** Optical rotation for serranticin in acetone.

| N | Average | Standard Deviation | Relative standard deviation (%) | Max | Min | T (C°) |
| --- | --- | --- | --- | --- | --- | --- |
| 5 | -26.40 | 2.97 | -11.25 | -22 | -30 | 20 |

**Supplementary Table S7 Identification of the bioactive compound serranticin.** ^1^H-NMR (600 MHz) and ^13^C-NMR (150 MHz) spectroscopic data for serranticin in DMSO-*d*_6_.

| Position | 1H-NMR (*J* in Hz) | 13C-NMR |
| --- | --- | --- |
| 1 |  | 110.49 |
| 2 | 12.52 (s) | 148.45 |
| 3 |  | 145.91 |
| 4 | 7.68 (dd, *J* = 7.9, 1.4) | 118.01 |
| 5 | 7.52 (t, *J* = 7.9) | 118.82 |
| 6 | 7.85 (dd,*J*= 7.9, 1.4) | 119.58 |
| 7 |  | 165.85 |
| 9 | 5.24 (d, *J* = 7.4) | 73.88 |
| 10 | 5.64 (m) | 78.97 |
| 12 | 2.23 (3H, d, *J* = 6.4) | 20.83 |
| 1' |  | 169.75 |
| 2' (NH) | 9.09 (t,*J*= 6.0) |  |
| 3' | 4.08 (2H, m) | 36.85 |
| 4' | 3.28 (2H, m) | 29.08 |
| 5' | 3.97 (2H, m) | 36.74 |
| 6' (NH) | 9.58 (t,*J* = 5.9) |  |
| 7' |  | 169.94 |
| 1'' |  | 115.09 |
| 2'' | 13.54 (s) | 149.92 |
| 3'' | 10.00 (2H, s, 3) | 146.42 |
| 4'' | 7.75 (dd,*J* = 7.9, 1.4) | 118.89 |
| 5'' | 7.45 (t,*J* = 8.0) | 118.08 |
| 6'' | 8.04 (dd, *J* = 8.0, 1.4) | 117.21 |

**Supplementary Table S8 Serranticin gene clusters.** Annotation of the serranticin biosynthetic cluster A.

| Gene | AA length | Predicted Function | Strain | Identity (%) | Similarity (%) |
| --- | --- | --- | --- | --- | --- |
| *src*A | 252 | 2,3-dihydro-2,3-dihydroxybenzoate dehydrogenase | *Serratia plymuthica* V4 | 85 | 100 |
| *src*B | 284 | Isochorismatase | *Serratia plymuthica* V4 | 88 | 99 |
| *src*C | 402 | Isochorismate synthase | *Serratia plymuthica* V4 | 86 | 100 |
| *src*E | 542 | Enterobactin synthase component E | *Serratia plymuthica* V4 | 87 | 100 |
| *src*F | 1314 | Enterobactin synthase subunit F | *Serratia plymuthica* V4 | 86 | 100 |
| *srcG* | 561 | Acetolactate synthase | *Serratia plymuthica* V4 | 88 | 100 |
| *src*I | 319 | Iron-enterobactin transporter periplasmic binding protein | *Serratia plymuthica* V4 | 84 | 100 |
| *src*J | 422 | Enterobactin transporter EntS | *Serratia plymuthica* V4 | 86 | 100 |
| *src*K | 357 | Ferric enterobactin transport system permease protein | *Serratia plymuthica* V4 | 87 | 100 |
| *src*L | 341 | Iron-enterobactin ABC transporter permease | *Serratia plymuthica* V4 | 87 | 100 |
| *src*M | 267 | Ferric enterobactin transport ATP-binding protein | *Serratia plymuthica* V4 | 88 | 98 |
| *src*N | 759 | TonB-dependent siderophore receptor | *Serratia plymuthica* V4 | 87 | 99 |
| *src*O | 456 | Enterochelin esterase | *Serratia plymuthica* V4 | 84 | 100 |
| *src*P | 535 | ABC transporter substrate-binding protein | *Serratia plymuthica* V4 | 88 | 98 |

**Supplementary Table S9 Serranticin gene clusters.** Annotation of the serranticin biosynthetic cluster B.

| Gene | AA length | Predicted Function | Strain | Identity (%) | Similarity (%) |
| --- | --- | --- | --- | --- | --- |
| *src*F1 | 533 | Glutamate racemase | *Serratia plymuthica* V4 | 80 | 99 |
| *src*F2 | 1031 | Non-ribosomal peptide synthetase | *Serratia plymuthica* V4 | 84 | 100 |
| *src*F3 | 974 | Condensation domain | *Serratia plymuthica* V4 | 81 | 100 |
| *src*H | 448 | Condensation domain | *Serratia plymuthica* V4 | 81 | 100 |
| *src*Q | 707 | TonB-dependent receptor | *Serratia plymuthica* V4 | 89 | 100 |
| *src*R | 532 | ABC transporter substrate-binding protein | *Serratia plymuthica* V4 | 88 | 99 |
